# Ultrasonic-responsive piezoelectric stimulation enhances sonodynamic therapy for HER2-positive breast cancer

**DOI:** 10.1186/s12951-024-02639-6

**Published:** 2024-06-25

**Authors:** Zhiguang Chen, Lizhi Yang, Zhimin Yang, Zihua Wang, Wen He, Wei Zhang

**Affiliations:** 1 Department of Ultrasound, Beijing Tiantan Hospital, Capital Medical University, Beijing, 100050 China; 2 CAS Key Laboratory for Biomedical Effects of Nanomaterials and Nanosafety, CAS Center for Excellence in Nanoscience, National Center for Nanoscience and Technology, Beijing, 100190 China; 3 Fujian Provincial Key Laboratory of Brain Aging and Neurodegenerative Diseases, School of Basic Medical Sciences, Fujian Medical University, Fuzhou, Fujian 350122 China

**Keywords:** HER2-positive breast cancer, Piezoelectric material, Sonodynamic therapy, Sonosensitizer, P(VDF-TrFE)

## Abstract

**Introduction:**

Breast cancer ranks second as the most common malignancy globally, after lung cancer. Among the various subtypes of breast cancer, HER2 positive breast cancer (HER2 BC)poses a particularly challenging prognosis due to its heightened invasiveness and metastatic potential. The objective of this study was to construct a composite piezoelectric nanoparticle based on poly(vinylidene fluoride-trifluoroethylene) (P(VDF-TrFE)) for imaging and treatment of HER2 BC.

**Method:**

By reshaping the crystal structure of P(VDF-TrFE) piezoelectric nanoparticles, improving hydrophilicity, and incorporating imaging capabilities, we developed piezoelectric composite nanoparticles (PGd@tNBs) that integrate imaging and therapeutic functions. The in vitro characterization encompassed the assessment of piezoelectric properties, hydrophilicity, imaging performance, and therapeutic efficacy of these particles. The targeting and therapeutic effectiveness of PGd@tNBs particles were further validated in the SK-BR3 cell line and subsequently confirmed in HER2-positive tumor-bearing mice.

**Results:**

The nanoparticle demonstrated excellent biocompatibility and impressive multimodal imaging performance. Magnetic resonance imaging (MRI) observations revealed significant accumulation of PGd@tNBs particles in the HER2 positive tumor, exhibiting superior contrast-enhanced ultrasound performance compared to traditional ultrasound contrast agents, and small animal in vivo imaging showed that PGd@tNBs particles were primarily excreted through respiration and urinary metabolism. Piezoforce Microscopy characterization highlighted the outstanding piezoelectric properties of PGd@tNBs particles. Upon targeted binding to HER2-BC, ultrasound stimulation influenced the cell membrane potential, leading to reversible electroporation. This, in turn, affected the balance of calcium ions inside and outside the cells and the mitochondrial membrane potential. Following ingestion by cells, PGd@tNBs, when exposed to ultrasound, triggered the generation of reactive oxygen species (ROS), resulting in the consumption of glutathione and superoxide dismutase and achieving sonodynamic therapy. Notably, repeated ultrasound stimulation, post PGd@tNBs particles binding and entry into cells, increased ROS production and elevated the apoptosis rate by approximately 45%.

**Conclusion:**

In conclusion, the PGd@tNBs particles developed exhibit outstanding imaging and therapeutic efficacy, holding potential for precise diagnosis and personalized treatment of HER2 BC.

**Supplementary Information:**

The online version contains supplementary material available at 10.1186/s12951-024-02639-6.

## Introduction

According to cancer registration data [1], breast cancer accounted for a substantial 11.6% of all cancer cases in 2022, it is second only to lung cancer as the most prevalent malignancy worldwide. Among various breast cancer subtypes, HER2-positive breast cancer (HER2 BC) constitutes approximately 20–30% of cases, exhibiting heightened invasiveness and a proclivity for metastasis [2]. The amplification of the HER2 gene, which leads to the overexpression of HER2 protein, this overexpressed HER2 protein activates a series of signaling pathways, promoting the proliferation, division, and diffusion of tumor cells, thereby enhancing the tumor’s invasiveness and metastatic capacity. Furthermore, cells in HER2-positive breast cancer often exhibit a high proliferation rate and a low apoptosis rate, this means that tumor cells can rapidly grow and spread, while being difficult to eliminate through natural apoptosis processes [3, 4]. The widespread use of trastuzumab has revealed that a considerable proportion of patients (66–88%) develop resistance to the treatment when administered as a monotherapy [5, 6]. This highlights the critical need for innovative therapeutic strategies to effectively address acquired resistance in HER2 BC.

Sonodynamic therapy (SDT) has emerged as a promising non-invasive approach for tumor treatment, offering advantages such as deep tissue penetration and cost-effectiveness. However, the therapeutic effectiveness of SDT is impeded by the hypoxic conditions within the tumor microenvironment (TME) [7, 8]. In recent years, piezoelectric materials have gained recognition as valuable sensitizers for SDT [9, 10]. Among these, poly (vinylidene fluoride-trifluoroethylene) (P(VDF-TrFE)) stands out as a notable organic piezoelectric material. Possessing exceptional piezoelectric properties, robust mechanical characteristics, and biocompatibility, P(VDF-TrFE) finds widespread applications in electroacoustics, ultrasound technology, and mechanical engineering [11–13]. Moreover, P(VDF-TrFE) piezoelectric films have exhibited the capability to enhance neuronal differentiation [14], accelerate wound healing [15], impede cell proliferation, and induce apoptosis [16] upon stimulation with ultrasound.

In 2022, Pucci et al. [17] pioneered the application of P(VDF-TrFE) nanoparticles in the treatment of glioblastoma cells. Their study showcased the remarkable piezoelectric potential generation of P(VDF-TrFE) nanoparticles when exposed to ultrasound, triggering pathways for apoptosis activation and anti-proliferation in cancer cells.

Ultrasound, as a real-time and portable imaging method, has been widely utilized, but its tissue resolution is relatively low [18]. On the other hand, magnetic resonance imaging (MRI) offers excellent tissue resolution but lacks in temporal resolution [19]. In recent years, researchers have addressed these limitations by incorporating gadolinium/iron ions onto nano-vesicles, enabling dual-modal imaging of MRI and ultrasound, thus overcoming the drawbacks associated with their individual use [20, 21].

Building upon this foundation, we put forth the hypothesis that P(VDF-TrFE) piezoelectric material, when subjected to ultrasound excitation, can serve as a valuable candidate for SDT. Furthermore, the electric charges generated through this stimulation hold the potential to augment the therapeutic effectiveness of SDT.

**Fig. 1:**
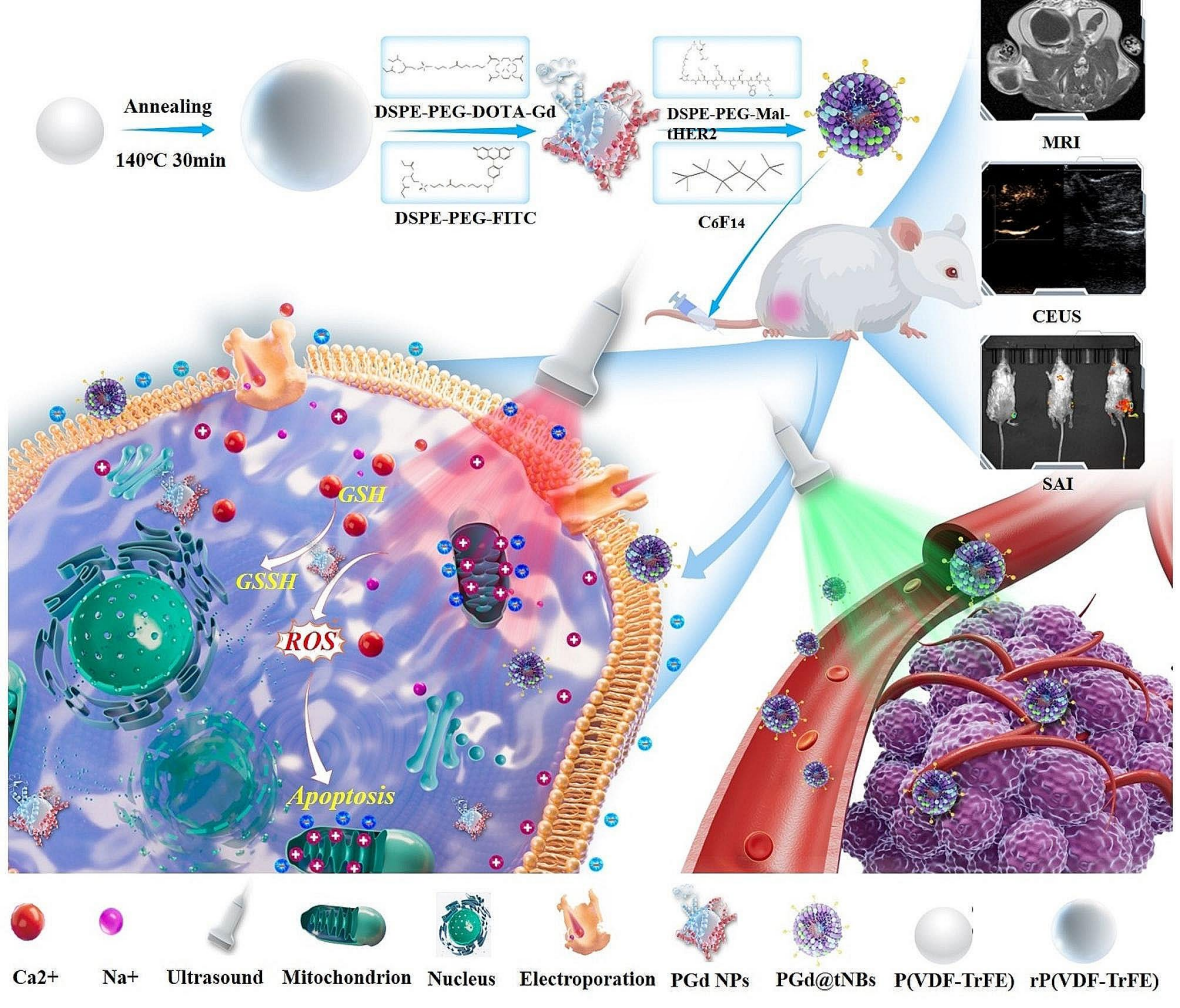
Schematic diagram of the synthetic process and antitumor mechanism of PGd@tNBs

Herein, we have successfully developed a novel HER2-targeting composite piezoelectric nanoparticle PGd@tNBs, which effectively combines both MRI imaging and contrast-enhanced ultrasound (CEUS) capabilities. In vitro and in vivo results indicate these nanoparticles have impressive specificity for HER2 BC, and upon ultrasound stimulation, generated charges that modulated the cell membrane potential. And then triggered an influx of calcium ions (Ca^2+^), ultimately leading to apoptosis by reducing mitochondrial membrane potential. Subsequent ultrasound stimulation of internalized nanoparticles resulted in a significant production of reactive oxygen species (ROS), contributing to further cellular damage. Thus, the PGd@tNBs particles not only exhibited biocompatibility as piezoelectric materials but also showcased remarkable SDT properties. Through the synergy of piezoelectric and sonodynamic therapies, these particles effectively suppressed the proliferation and induced apoptosis of SK-BR3 cancer cells (Fig. 1).

## Experimental methods

### Materials

P(VDF-TrFE) (70:30 mol %) was purchased from the Arkema platform with a purity of > 96%; 1, 2-Dipalmitoyl-sn-glycero-3-phosphorylcholine (DPPC), 1, 2-distearoyl-sn-glycero-3-phosphoethanolamine-N-[maleimide(polyethyleneglycol)] (DSPE-PEG-Mal), 1, 2-distearoyl-sn-glycero-3-phosphoethanolamine-poly(ethylene glycol)-Fluorescein (DSPE-PEG-FITC), and Cholesterol (Chol) were purchased from Shanghai Pengsheng BioTech Co., Ltd., China; 1, 2-distearoyl-sn-glycero-3-phosphoethanolamine-poly(ethylene glycol)-DOTA (DSPE-PEG-DOTA) was purchased from Xi’ an Ruixi Biotechnology Co., Ltd., China; FluoVoltTM assay kit was purchased from Thermofisher; Calcium ion fluorescent probe (Fluo-3 AM), Superoxide Dismutase (SOD) assay kit, Glutathione (GSH) detection kit, JC-1 assay kit, Calcein-AM/PI assay kit, and ROS detection kit were purchased from Yisheng Biotechnology (Shanghai) Co., Ltd., China. BALB/c mice purchased from Weitong Lihua, 16–20 g/mouse, female.

### Synthesis of nanoparticles

In our study, we opted for the thin film hydration-acoustic vibration method. In a succinct overview, the process involves multiple stages: Crystalline Phase Reshaping: P(VDF-TrFE) undergoes heat annealing at 140 °C, transforming it into reshaped P(VDF-TrFE), denoted as rP(VDF-TrFE). Gadolinium acetate and DSPE-PEG-DOTA solutions are meticulously combined in precise ratios, with pH adjustment to 7.4. This mixture is allowed to react overnight on a shaker. Subsequent steps encompass centrifugation and purification, leading to the isolation of DSPE-PEG-DOTA-Gd. PGd NPs Nanoparticles: The compounds DSPE-PEG-DOTA-Gd and DSPE-PEG-FITC are brought together in specific proportions and dissolved in the rP(VDF-TrFE) solution. The solution undergoes rotary evaporation to remove organic solvent. The resulting residual solution is then subjected to centrifugation and purification, culminating in purified PGd NPs nanoparticles. A targeting peptide containing the HER-2 targeting sequence CKLRLEWNR is synthesized through a condensation reaction. This synthesized peptide and DSPE-PEG-Mal solution are amalgamated in precise ratios, yielding DSPA-PEG-Mal-tHER2 through Michael addition click reaction.

Lipid Bilayer Formation: In this step, DPPC, Chol, and DSPE-PEG-Mal-tHER2 are skillfully combined in specific proportions. This amalgamation is employed to create a lipid bilayer via rotary evaporation.

Nanoparticle Formation: The PGd NPs solution, along with perfluorohexane and glycerol, is meticulously added to the lipid bilayer in precise ratios. This composite solution is dissolved at 60 °C and subsequently subjected to oscillation via a tip ultrasonicator, all while maintaining an ice bath environment. The solution resulting from oscillation undergoes differential centrifugation. The pellet obtained from the second centrifugation is subjected to thorough washing, resulting in the ultimate product: PGd@tNBs composite piezoelectric nanoparticles.

### Crystalline characterization of nanoparticles

The structural characteristics of both P(VDF-TrFE) and rP(VDF-TrFE) were elucidated through X-ray diffraction analysis (XRD). This technique enabled the identification of the respective crystalline phases present in the materials. The phase transitions of P(VDF-TrFE) and rP(VDF-TrFE) were further investigated using Raman imaging, providing insight into their compositional and conformational changes. The piezoelectric properties, including coefficients and potential, of both P(VDF-TrFE) and rP(VDF-TrFE) were rigorously examined utilizing Piezoresponse Force Microscopy (PFM). This microscopy-based technique enabled the quantification of their piezoelectric response at the nanoscale. The elemental composition and oxidation states of the constituents within PGd@tNBs were meticulously determined employing X-ray Photoelectron Spectroscopy (XPS). This advanced technique facilitated the characterization of the chemical bonding and surface composition of the nanoparticles, shedding light on their atomic-scale features. The particle size distribution and surface charge (ζ potential) of PGd@tNBs were analyzed using Dynamic Light Scattering (DLS). This method yielded critical information about the particle size distribution and surface properties, aiding in understanding their stability and behavior in solution.

### Morphological characterization of nanoparticles

The structural features of the lipid shell, PGd NPs, and PGd@tNBs were examined through Scanning Electron Microscopy (SEM) and Transmission Electron Microscopy (TEM). These high-resolution imaging techniques provided visual insights into the surface characteristics and internal structures of the materials at the micro- and nanoscale levels. Furthermore, the element composition and distribution of PGd@tNBs were investigated using Energy-Dispersive X-ray Spectroscopy (EDS) in conjunction with SEM. This allowed for the identification and mapping of the elemental constituents present within the nanoparticles, offering valuable information about their chemical composition and spatial arrangement. The morphological details and the distribution of green fluorescence in PGd@tNBs were analyzed utilizing Confocal Laser Scanning Microscopy (CLSM). This advanced microscopy technique facilitated the visualization of the particles’ structure and fluorescence emission patterns, providing crucial insights into their morphology and functional properties.

### Imaging performance of nanoparticles

The in vitro ultrasound imaging characterization of PGd@tNBs was conducted using a self-assembled setup. The setup involved suspending an intravenous drip bag at a predetermined height and introducing various solutions, including ddH_2_O (serving as a negative control), different concentrations of PGd@tNBs (ranging from 0.1 mg/mL to 0.5 mg/mL), and the Sonovue contrast agent (used as a positive control). An 18 MHz probe was selected for the imaging procedure, and imaging parameters were adjusted, with a gain set at 70% and a mechanical index of 0.08. For the in vitro MRI imaging characterization of PGd@tNBs, the approach focused on quantifying the concentration of gadolinium (Gd). This involved setting up different Gd concentrations at levels of 0.1 mM, 0.2 mM, 0.3 mM, 0.4 mM, and 0.5 mM. The negative control consisted of ddH_2_O, while the positive control employed DTPA-Gd. The in vitro Second Harmonic Generation (SAI) imaging characterization of PGd@tNBs was accomplished by imaging samples with varying concentrations: 0.1 mg/mL, 0.2 mg/mL, 0.3 mg/mL, 0.4 mg/mL, and 0.5 mg/mL. The fluorescence intensity for each concentration was quantified using Image J software.

### In vitro therapeutic characterization of nanoparticles

The assessment of PGd@tNBs particles’ ability to generate ROS under ultrasound excitation involved several experiments using different solutions:

Methylene Blue Solution Test for ROS Generation: different concentrations of PGd@tNBs solution were mixed with a fixed amount of methylene blue solution. The mixture was allowed to reach adsorption equilibrium for 1 h. Ultrasound stimulation was applied to the mixture. After ultrasound stimulation, the solution was centrifuged, and the upper layer was analyzed using a UV/Visible/NIR spectrophotometer. The goal was to observe the ultraviolet absorption band at 655 nm, indicative of ROS generation.

Benzoic Acid Solution Test for ·OH Radical Generation: different concentrations of PGd@tNBs solution were mixed with a specific amount of benzoic acid solution. Ultrasound stimulation was applied to the mixture. After ultrasound stimulation, the solution was centrifuged, and the upper layer was analyzed using a fluorescence spectrophotometer. The aim was to observe the fluorescence band within the range of 400–550 nm, indicating ·OH radical generation.

GSH and 2-Nitrobenzoic Acid Test for GSH Consumption: various concentrations of PGd@tNBs solution were combined with glutathione and 2-nitrobenzoic acid. Ultrasound stimulation was applied to the mixture. The solution was then analyzed using a UV/Visible/NIR spectrophotometer. The purpose was to observe the ultraviolet absorption band at 412 nm, which signifies changes in glutathione concentration due to ultrasound-induced reactions.

### Targeting ability of nanoparticles to HER2 positive cells

PGd@tNBs nanoparticles were co-incubated with cells for a short period (≤ 5 h). Cell membrane targeting was observed using Dil staining to assess the amount of particles targeting the cell membrane. The interaction between PGd@tNBs nanoparticles and cells was examined by incubating them together on silicon substrates, followed by SEM imaging after gradient dehydration to observe the binding between the nanoparticles and cells. The cytotoxic effects of PGd@tNBs and non-targeted PGd@NBs were studied using a CCK-8 assay on HER-2 positive cells (SK-BR3) and HER-2 negative cells (MCF-7). Subsequently, flow cytometry analysis was conducted to quantify the binding of PGd@tNBs nanoparticles to SK-BR3 cells at various time points (0.5 h, 1 h, 2 h, 3 h, 4 h, 5 h) after co-incubation.

### The sonodynamic therapy ability of nanoparticles

Extended Co-Incubation and Nanoparticle Entry Determination: PGd@tNBs nanoparticles were co-incubated with cells for an extended period, typically ranging from 6 to 72 h. Lysosome staining was performed to determine the timing of nanoparticle entry into cells. This staining technique utilizes specific dyes that accumulate in lysosomes, helping to identify the time point at which nanoparticles are internalized by cells. Flow cytometry analysis was employed to quantitatively assess the internalization of PGd@tNBs nanoparticles by cells over the extended co-incubation period. This technique allows for the measurement of fluorescence signals emitted by labeled nanoparticles within cells, indicating their internalization.

Cell Experiments and Ultrasound Treatment Groups: The study design involved categorizing cell experiments into six distinct groups based on different ultrasound treatment times. The groups included: Control group, US1 group, US2 group, PGd@tNBs only group, PGd@tNBs + US1 group, and PGd@tNBs + US2 group. The goal was to investigate the effects of different treatment strategies involving ultrasound stimulation, nanoparticles, and their combinations on cellular responses.

Visualization of Intracellular Calcium Ion Levels: The intracellular calcium ion levels were visualized using a fluorescence probe called Fluo-3 AM. Fluo-3 AM is a calcium-sensitive dye that becomes fluorescent upon binding to calcium ions, allowing for the visualization of changes in intracellular calcium levels. The purpose was to assess the influence of various treatments on intracellular calcium ion concentration.

ROS Generation and Temporal Variations Analysis: The generation of ROS and its temporal variations were assessed using a ROS detection assay kit. ROS detection assays involve using specific fluorescent probes that can react with ROS molecules, producing a fluorescence signal proportional to ROS levels. This experiment aimed to determine how different treatments influenced ROS production over time.

Intracellular GSH and SOD Measurement: The consumption of intracellular GSH and SOD in each treatment group was measured using GSH and SOD detection assay kits. These assays provide insights into the oxidative stress and antioxidant defense mechanisms within cells in response to the various treatments.

### In vitro electrical stimulation therapy of nanoparticles

JC-1 staining was performed using CLSM to assess mitochondrial membrane potential under different treatment conditions. Cells were stained with JC-1 dye, and excitation wavelengths of 485 nm and 535 nm were set to evaluate mitochondrial membrane potential based on red and green fluorescence signals. Quantitative assessment of mitochondrial membrane potential was conducted using the FL2 and FL1 channels in flow cytometry. For PGd@tNBs + US group and Control group cells, cell membrane potential was measured using the Fluo Volt ^TM^ cell membrane potential probe. Continuous imaging was performed using CLSM, and subsequent quantification of fluorescence intensity changes was conducted using Image J. Flow cytometry was employed to quantitatively analyze the proportions of early apoptosis, late apoptosis, and necrosis in each group of cells. The Calcein-AM/PI staining assay was used to observe the viability of cells after different treatments. The effects of different treatments on the proliferation, migration, and invasion of SK-BR3 cells were assessed through wound healing and Transwell invasion assays.

### Multimodal imaging ability of nanoparticles in vivo

After inducing anesthesia with isoflurane, the tumor-bearing mice were immobilized on the operating table and maintained under continuous anesthesia via a facial mask. For ultrasound imaging, the ultrasound imaging mode was initiated, and appropriate imaging parameters were adjusted. A 200 µL solution of PGd@tNBs was intravenously injected through the tail vein. Real-time scanning was performed to visualize and record the perfusion status of the tumor area. For MRI, the mice were positioned within the coil under anesthesia via a facial mask. Before administering the contrast agent, a location scan was performed for positioning. Subsequently, T1-weighted and T1 mapping scans were conducted. Following the scans, a 200 µL solution of PGd@tNBs was administered via the tail vein. Imaging was then conducted at 1 h, 3 h, 6 h, and 24 h post-administration at the same location. In the case of Small Animal In Vivo Imaging (SAIVI), after intravenously injecting a 200 µL solution of PGd@tNBs, the mice were placed in the SAI imaging area for imaging.

### Biosafety of nanoparticles in vivo

During the treatment of tumor-bearing mice, several procedures were carried out to monitor the health and assess potential toxic effects. Blood Sample Collection for Complete Blood Count (CBC) Analysis: Blood samples were collected from the mice using the retro-orbital venous puncture method. Samples were collected on the 1st, 3rd, and 5th days of treatment. The collected blood samples were subjected to CBC analysis. CBC analysis provides information about the different types of blood cells, including red blood cells, white blood cells, and platelets.

Blood Sample Collection for Biochemical Analysis: On the final day of treatment, a larger blood sample (at least 200 µL) was collected from the mice via retro-orbital venous puncture. The collected blood was then subjected to centrifugation to separate the serum from the other components. The upper serum layer was used for biochemical analysis. Biochemical analysis involves assessing various parameters such as liver enzymes (AST, ALT), kidney function markers, and other relevant indicators in the serum to evaluate the overall health of the mice and potential effects of treatments.

Euthanization and Organ Collection: At the end of the treatment period, the mice were euthanized using cervical dislocation. Major organs including the heart, liver, spleen, lung, and kidney were collected for further analysis. These organs were chosen because they are crucial indicators of overall health and potential toxic effects.

Hematoxylin and Eosin (HE) Staining: The collected organ tissues were subjected to HE staining. HE staining is a common histological technique that allows for the visualization of cellular structures and morphology in tissues. The stained tissue sections were observed under a microscope to identify any potential toxic effects on the major organs due to different treatments.

### The therapeutic effect of nanoparticles on tumors in vivo

During the in vivo treatment of the tumor-bearing mice, the following procedures were conducted to evaluate the treatment effectiveness and analyze the tumor tissue:

Routine Ultrasound Scans: Ultrasound scans were performed on the tumor area both at the beginning and at the end of the treatment period. Different blood flow imaging techniques, including Superb Microvascular Imaging (SMI), Tissue Doppler Imaging (TDI), Power Doppler Imaging (PDI), and Color Doppler Imaging, were utilized to assess blood flow characteristics within the tumor. Elastic imaging techniques, such as Real-Time Tissue Elastography and Shear Wave Elastography, were employed to evaluate the elasticity or stiffness of the tumor tissue.

Tumor Tissue Collection and Processing: After completing the treatment, a portion of the tumor tissue was rapidly frozen at -80 °C for future analysis. This frozen tissue was preserved to study the levels of ROS as well as for other potential analyses. Another portion of the tumor tissue was fixed by immersing it in a 4% paraformaldehyde solution for further processing and histological analysis.

Histological Analysis: The fixed tumor tissue was subjected to HE staining. HE staining allowed for the examination of tissue morphology and structural changes at a cellular level, providing insights into potential treatment effects and any alterations in tissue organization.

Transferase dUTP nick end labeling (TUNEL) assay and Ki-67 Staining: The fixed tumor tissue was used for the Terminal deoxynucleotidyl TUNEL assay. The TUNEL assay is utilized to detect apoptotic cells by labeling the DNA fragments generated during apoptosis. Ki-67 staining was also performed on the tissue sections to assess the proliferation status of cells. Ki-67 is a marker for cell proliferation.

ROS and Immunofluorescence Analysis: The frozen tumor tissue was analyzed to measure the levels of ROS, which can indicate oxidative stress and cellular damage. Immunofluorescence analysis could be performed to detect specific markers or proteins associated with oxidative stress, apoptosis, or other cellular processes.

## Results and discussions

### Characterization of the PGd@tNBs nanoparticles

**Fig. 2:**
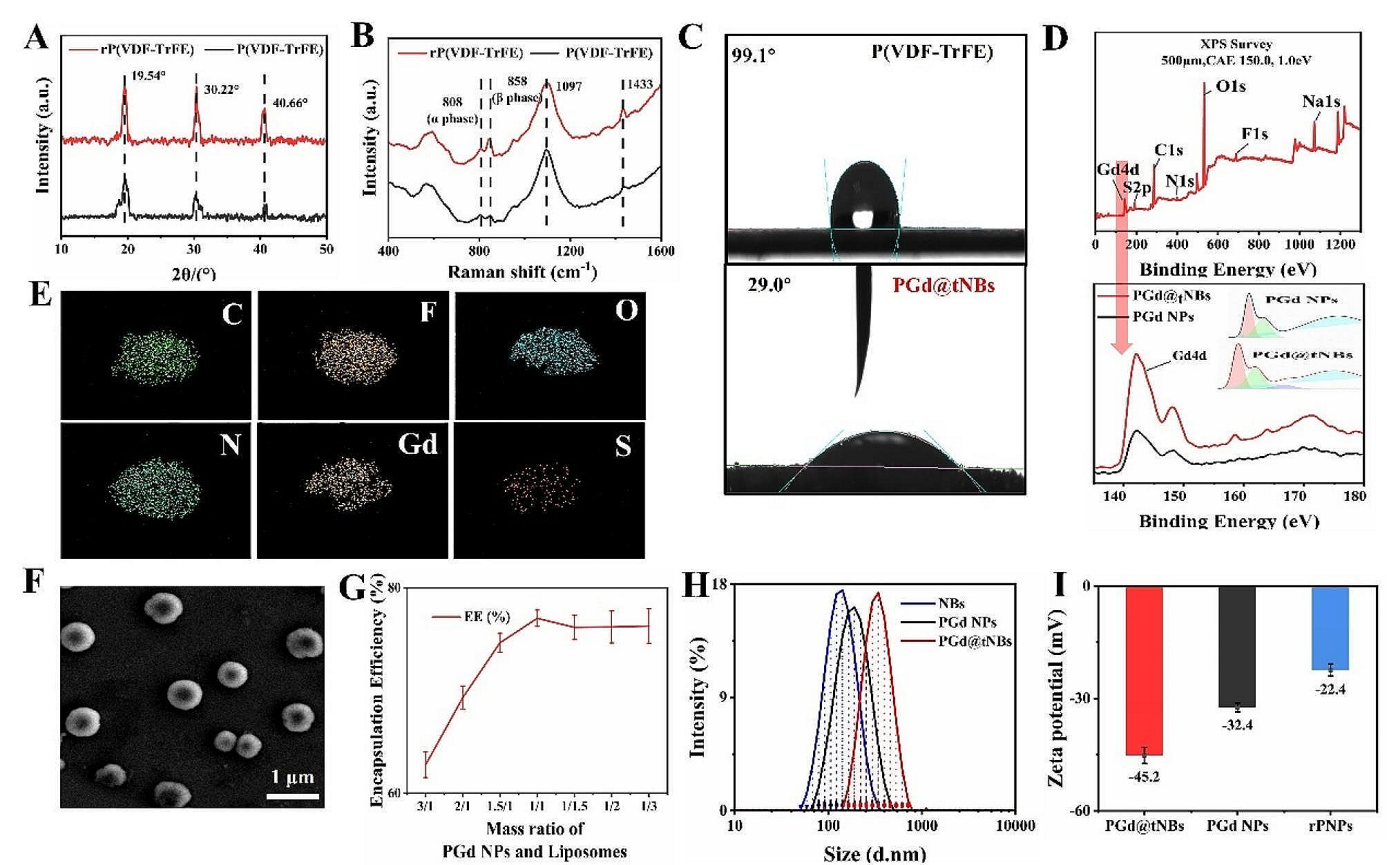
Characterization of PGd@tNBs nanoparticles. **(A)** XRD results. **(B)** Raman spectroscopy results. **(C)** Contact angle measurement of P(VDF-TrFE) nanoparticles and PGd@tNBs. **(D)** XPS analysis of PGd@tNBs (upper), valence state changes of Gd (below). **(E)** EDS analysis of PGd@tNBs nanoparticles. **(F)** SEM of PGd@tNBs nanoparticles. **(G)** Encapsulation efficiency of PGd@tNBs nanoparticles. **(H)** Particle size distribution of PGd@tNBs nanoparticles. **(I)** Zeta potential of PGd@tNBs nanoparticles

The technique employed to alter the crystalline phase of P(VDF-TrFE) particles was adapted from prior research [17, 22, 23]. In this studies, thermal annealing induced a transition from the ferroelectric phase to the paraelectric phase within the P(VDF-TrFE) material [24]. When subjected to a temperature of 140 °C, the X-ray diffraction (XRD) peak intensity of rP(VDF-TrFE) particles exhibited a significant increase (Fig. 2A). This enhancement was attributed to lattice expansion, leading to an augmented interchain distance. Consequently, the mobility of C-F dipoles within the crystal was improved, and friction forces were reduced [25, 26]. In the realm of Raman imaging, the peak at 858 cm^-1 corresponded to the β phase, whereas the peak at 808 cm^-1 corresponded to the α phase [27, 28]. Upon the alteration of the crystalline phase, there was a notable increase in the intensity of the β peak. Consequently, there was a substantial elevation in the ratio of β to α peak intensities (from 1.3 to 2.5) (Fig. 2B). The restructuring of copolymer chains due to annealing led to an expansion of the intermolecular spacing. This, in turn, weakened interchain interactions and minimized conformational defects [27, 29]. As a result, there was a pronounced improvement in the stability of molecular chains, as represented by the intensity of the 1433 cm^-1 peak.

Through the surface coating of the P(VDF-TrFE) materia to enhance hydrophilicity [17, 30], a notable reduction in the contact angle of PGd@tNBs particles is achieved, measuring only 29.0° after the enhancement process, while the contact angle of P(VDF-TrFE) is 99.1° (Fig. 2C). The hydrophilicity was increased by adding DSPE-PEG-DOTA-Gd to provide MRI imaging capability and DSPE-PEG-FITC to give fluorescent properties. Consequently, XPS elemental analysis divulges the presence of elements such as Gd, S, N, and O, in addition to C and F elements (Figure S1). Following the introduction of DSPE-PEG-Mal-tHER2 and C_6_F_14_, there is no substantial alteration in the elemental composition. However, as the reaction progresses, a discernible chemical shift occurs in the Gd ions within PGd@tNBs particles. This shift is manifested by an increase in their valence electrons from + 3 in PGd NPs particles to + 4 in PGd@tNBs (Fig. 2D). Simultaneously, there is a modification in the valence energy of carbon elements as well (Figure S2). Furthermore, EDS results substantiate the homogeneous distribution of oxygen, nitrogen, and gadolinium elements on the surface of PGd@tNBs particles (Fig. 2E). The PGd@tNBs particles displayed a relatively dispersed solid liposome structure under SEM (Fig. 2F). Further, a more intuitive view of the liposomes located on the surface of the nanoparticles was characterized by TEM (Figure S3).

Upon increasing the mass ratio of PGd NPs particles to the lipid shell from 3:1 to 1:1, the encapsulation efficiency of PGd@tNBs particles reaches approximately 77% (Fig. 2G). The pure lipid shell exhibits a diameter of approximately 110 nm, while the particle size of PGd NPs measures around 150 nm. Following sonication, the size of PGd@tNBs particles is roughly 250 nm (Fig. 2H), and polydispersity index (PDI) of PGd@tNBs particles is 0.223 (Figure S4a). Intriguingly, the absolute value of ζ-potential for PGd@tNBs particles is the highest (Fig. 2I), thus accounting for their stable particle size maintained at approximately 270 nm during storage for 1 to 3 weeks at 4 °C (Figure S4b). Additionally, our separate study has also corroborated that following crystal structure remodeling, the P(VDF-TrFE) particles exhibit stabilized particle sizes of around 200 nanometers, particularly in the DMEM culture medium, owing to their enhanced hydrophilicity [31].

### The imaging ability of PGd@tNBs nanoparticles was characterized in vitro

**Fig. 3:**
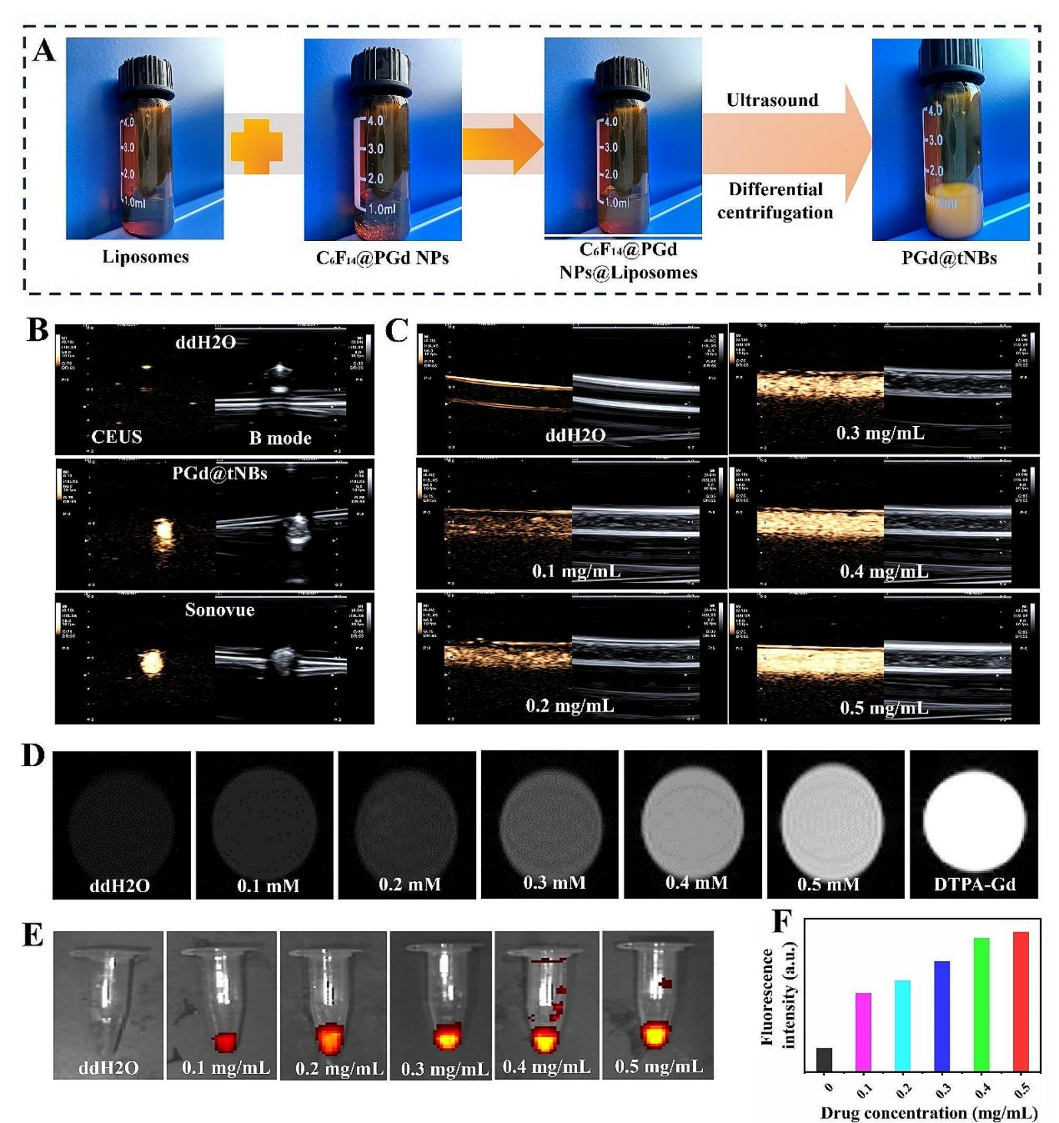
The imaging ability of PGd@tNBs nanoparticles was characterized in vitro. **(A)** Schematic representation of the synthesis process of PGd@tNBs nanoparticles. **(B)** Ultrasound imaging comparison between PGd@tNBs nanoparticles and Sonovue contrast agent. **(C)** Ultrasound imaging efficiency of PGd@tNBs nanoparticles at different concentrations. **(D)** MRI imaging capability comparison of PGd@tNBs nanoparticles at different concentrations with DTPA-Gd contrast agent. **(E)** Fluorescence imaging capability of PGd@tNBs nanoparticles at different concentrations. **(F)** Quantitative analysis of fluorescence intensity

Utilizing the HER2-targeting peptide (sequence: KLRLEWNR) previously chosen by our research team [32] (Figure S5), we employed a click chemistry reaction to facilitate the conjugation of the HER2-targeting peptide with DSPE-PEG-Mal. Subsequently, this conjugate was combined with DPPC and Chol to form the lipid shell (Figure S6). With the introduction of PGd NPs and C_6_F_14_, the resultant mixture underwent ultrasonic oscillation, leading to the generation of PGd@tNBs particles (Fig. 3A).

In comparison to conventional contrast agents, nanoscale ultrasound contrast agents offer notable advantages in terms of biocompatibility, serum stability, and extended lifespan. Presently, they find utility across various domains, including musculoskeletal imaging [33], vascular plaque imaging [21, 34], and assessment of ablative therapy [35]. Utilizing Sonovue as the positive control group and double-distilled water as the negative control, our synthesized PGd@tNBs nanoparticles exhibit robust CEUS capabilities (Fig. 3B), with their imaging performance enhanced proportionately as the particle concentration increases (Fig. 3C). Gadolinium-based chelates, renowned for their capability to shorten T1 relaxation times, are widely utilized in MRI imaging [36]. The effectiveness of imaging is closely linked to the concentration of gadolinium ions [36, 37]. In our investigation, although the MRI imaging performance of PGd@tNBs particles with a gadolinium ion concentration of 0.5 mM falls short of that observed with a pure DTPA-Gd solution, it still suffices to generate contrast against the control group (Fig. 3D). The r1 relaxivity of PGd@tNBs were 2.24 mM^− 1^s^− 1^. Furthermore, the fluorescence intensity of PGd@tNBs particles demonstrates an upward trend with increasing concentrations (Fig. 3E and F). These findings highlight the potential utility of PGd@tNBs particles as a multimodal contrast agent, such as MRI, ultrasound imaging or fluorescence imaging.

### Multimodal imaging of PGd@tNBs nanoparticles in vivo

**Fig. 4:**
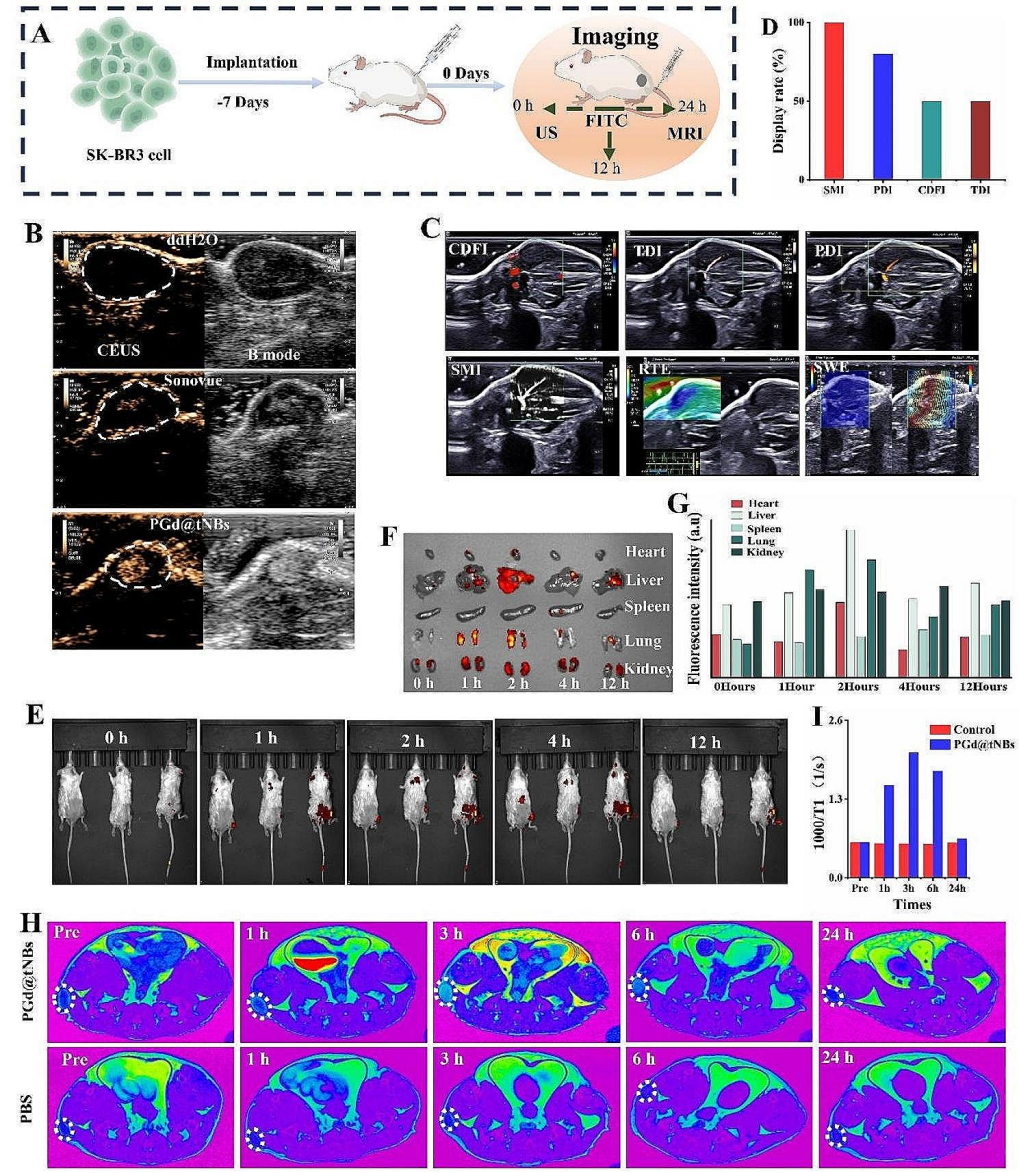
Multimodal imaging of PGd@tNBs nanoparticles in vivo. **(A)** Schematic representation of Balb/c mice tumor induction and imaging. **(B)** Ultrasound imaging of PGd@tNBs nanoparticles. **(C)** Ultrasound blood flow imaging and ultrasound elastography of mouse tumors. **(D)** Ultrasound blood flow imaging display rate. **(E)** In vivo fluorescence imaging of PGd@tNBs nanoparticles. **(F)** Distribution of PGd@tNBs nanoparticles in organs at different times. **(G)** Quantitative assessment of fluorescence intensity in mouse organs. **(H)** MRI of PGd@tNBs nanoparticles. **(I)** Quantitative analysis of T1 relaxation times in MRI imaging

Figure 4A depicts the schematic detailing tumor implantation and imaging in mice. Following the intravenous injection of a 200 µL solution containing PGd@tNBs nanoparticles at a concentration of 300 µg/mL, a swift enhancement in the tumor area was observed. Compared to the traditional contrast agent, Sonovue, the PGd@tNBs nanoparticles exhibit a more extensive and prolonged distribution within tumor tissue (Fig. 4B and Figure S7), this is attributed to the fact that nanoscale ultrasonic contrast agents typically possess superior stability, enabling them to circulate in the body for a prolonged duration without being eliminated by the immune system. Moreover, their minute size allows for easier infiltration into the extravascular space of tumor tissue, and potentially accumulation in the tumor tissue through the enhanced permeability and retention (EPR) effect [38]. Furthermore, we have employed HER2-targeted modifications on their surface, which enables these nanoparticles to actively target tumor cells. Subsequently, we executed standard ultrasound scans on the tumor, capturing Color Doppler Flow Imaging (CDFI), Tissue Doppler Imaging (TDI), Power Doppler Imaging (PDI), and Superb Microvascular Imaging (SMI) on the same cross-section of the tumor (Fig. 4C). Notably, SMI yielded a 100% success rate in its visualization (Fig. 4D). This achievement can be attributed to SMI’s specialized algorithm, adept at effectively discerning microvascular flow from motion artifacts and other noise artifacts, thus enabling users to seamlessly visualize the microvascular system [39].

Upon intravenous injection of the PGd@tNBs solution, robust fluorescence signals were observed to accumulate within the tumor area after 2 h, even after 12 h, a faint fluorescent signal can still be observed at the tumor tissue (Fig. 4E). It is worth noting that, 2 h after injection of the PGd@tNBs solution, the fluorescent signals in the liver, lungs, and kidneys further intensified. After 12 h, a slight residual fluorescence signal was still detected in the kidneys of the mice, suggesting that the PGd@tNBs particles were likely metabolized via urine excretion (Fig. 4F). Throughout the entire observation period (12 h), the fluorescent signal in the kidneys remained relatively high (Fig. 4G). After the intravenous administration of PGd@tNBs, enhancement within the vicinity of the tumor area commenced at 1 h, intensified at 3 h, began to diminish at 6 h, and by the 24-hour mark, the tumor tissue enhancement had entirely subsided, causing the tumor T1 relaxation time to revert to a level akin to that preceding injection (Fig. 4H). Conversely, the tumor tissue T1 relaxation time within the PBS group exhibited no discernible alteration throughout (Fig. 4I).

### Effect of PGd@tNBs particles on membrane potential upon ultrasound stimulation

**Fig. 5:**
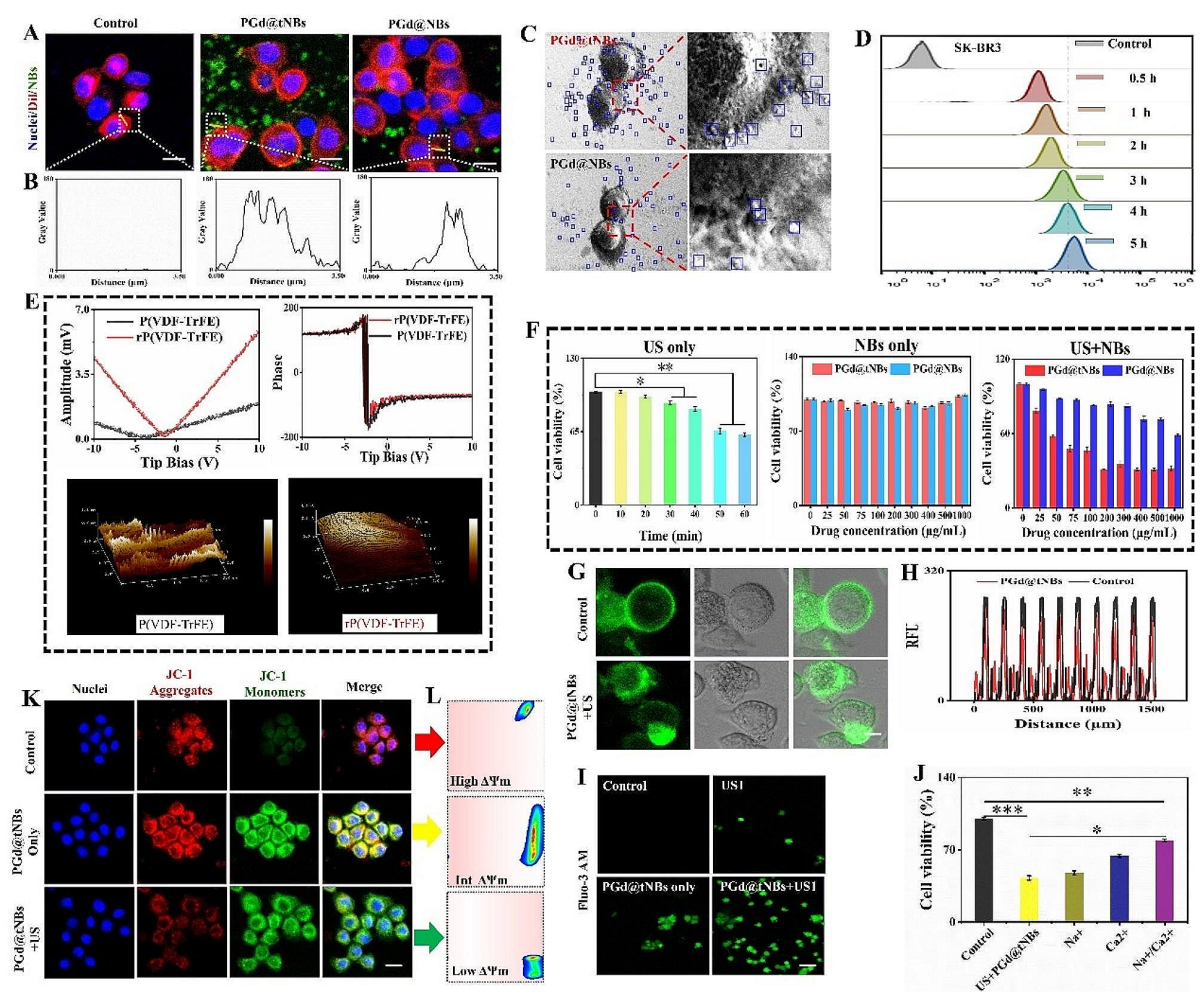
Effect of PGd@tNBs particles on membrane potential upon ultrasound stimulation. **(A)** CLSM characterization of PGd@tNBs nanoparticle targeting to cell membranes (Scale bar, 20 μm). **(B)** Quantitative analysis of red cell membrane and green PGd@tNBs nanoparticle fluorescence. **(C)** SEM characterization of PGd@tNBs and PGd@NBs binding to SK-BR3 cells. **(D)** Uptake of PGd@tNBs nanoparticles by SK-BR3 cells within a short period (≤ 5 h). **(E)** Hysteresis loop, polarization-field loop, and surface potential of rP(VDF-TrFE) and P(VDF-TrFE). **(F)** Effects of ultrasound stimulation alone or PGd@tNBs nanoparticles alone and PGd@tNBs nanoparticles under ultrasound irradiation on the activity of SK-BR3 cells (*n* = 3). **(G)** Measurement of cell membrane potential using FluoVolt™ dye (Scale 3 μm). **(H)** Quantitative analysis of cell membrane fluorescence intensity. **(I)** Intracellular free calcium ion characterization using Fluo-3AM calcium ion probe (Scale 10 μm). **(J)** Impact of calcium ion channel blockers and sodium ion channel blockers on cell viability (*n* = 3). **(K)** CLSM characterization of mitochondrial membrane potential (Scale 5 μm). **(L)** Quantitative analysis of mitochondrial membrane potential

Both PGd@tNBs and non-targeted PGd@NBs nanoparticles were separately incubated with SK-BR3 cells for a duration of 3 h. A pronounced dispersion of PGd@tNBs green fluorescence was observed surrounding the cancer cells, whereas PGd@NBs particles were relatively sparse (Fig. 5A and B). Furthermore, we turned to SEM to visually characterize the interaction between nanoparticles and cells. The images clearly indicated a significantly higher count of PGd@tNBs nanoparticles binding to SK-BR3 cells compared to PGd@NBs nanoparticles (Fig. 5C). Notably, the uptake of PGd@tNBs nanoparticles to SK-BR3 cells escalated as the incubation duration was extended within a short timeframe (≤ 5 h) by flow cytometry (Fig. 5D). The phase hysteresis loop and amplitude hysteresis loop serve as the most straightforward indicators of the piezoelectric response. Upon applying an external electric field of 10 V, there is a noticeable increase in the surface potential of rP(VDF-TrFE), this increase is accompanied by a more pronounced variation in amplitude, and the particle surface morphology becomes more regular (Fig. 5E). Through the estimation of the maximum amplitude of the piezoelectric signal, the effective piezoelectric coefficient *d*_33_ of rP(VDF-TrFE) is approximately − 8.8 pC/N (Figure S8). Following this, we explored the heightened cytotoxic effect of nanoparticles with crystal restructuring under ultrasound stimulation on SK-BR3 cells. This effect was observed under conditions where individual ultrasound stimulation or nanoparticles alone had no impact on cell viability (Fig. 5F and Figures S9–S12). Continuing, we noted a decrease in the fluorescence signal intensity of the cell membrane under ultrasound exposure (Fig. 5H), indirectly indicating damage to the cell membrane potential (Fig. 5G and Figures S13). In the presence of ultrasound, PGd@tNBs particles have the capacity to elevate the concentration of intracellular free Ca^2+^ within cancer cells (Fig. 5I).

Increased levels of intracellular Ca^2+^ can precipitate alterations in cell membrane potential, disrupt cell homeostasis, exert influence over cell proliferation, gene expression, ROS generation, and even trigger mitochondrial autophagy [40–42]. Interestingly, even in the presence of both calcium ion channel blockers and sodium ion channel blockers, cell viability was still significantly impaired. This suggests that PGd@tNBs nanoparticles, under ultrasound exposure, induce electroporation in cells, thereby promoting the influx of calcium ions (Fig. 5J). Distinct treatments can elicit changes in mitochondrial membrane potential, a phenomenon that can be visualized through the fluorescence signal emitted by the JC-1 dye [43]. In the control group, a substantial mitochondrial membrane potential was evident, causing the JC-1 dye to aggregate and emit red fluorescence. Conversely, the PGd@tNBs + US group experienced notable damage to the mitochondrial membrane potential, leading the JC-1 dye to exist in its monomeric form and emit green fluorescence (Fig. 5K). Subsequently, flow cytometry was employed to corroborate the alterations in membrane potential across the control group, solitary PGd@tNBs treatment, and PGd@tNBs + US treatment conditions (Fig. 5L).

### The piezoelectric effect of PGd@tNBs nanoparticles boost SDT

**Fig. 6:**
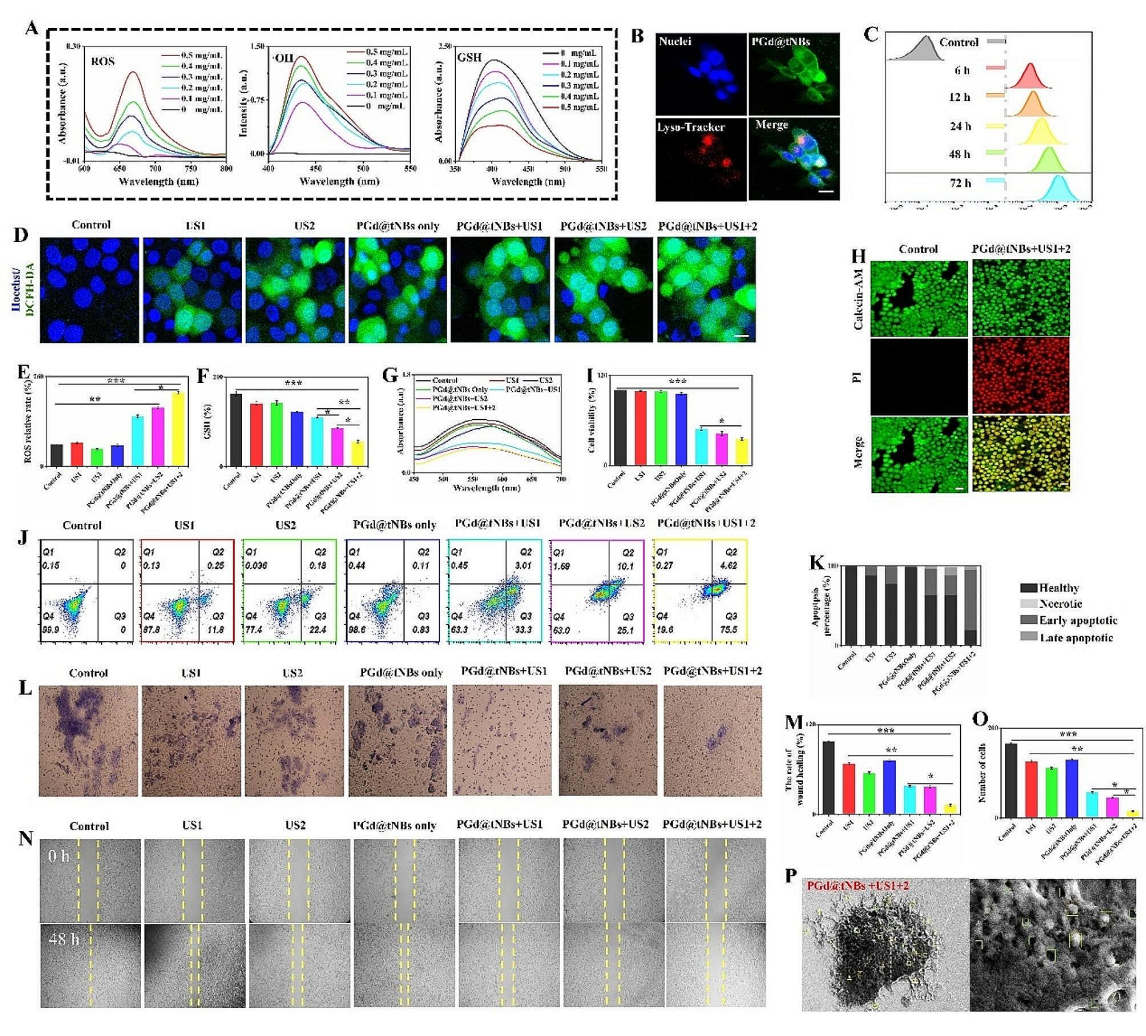
The piezoelectric effect of PGd@tNBs nanoparticles assists SDT. **(A)** The in vitro SDT efficacy of PGd@tNBs nanoparticles was characterized, the assessment included the generation ability of ROS and ·OH, as well as the consumption of glutathione. **(B)** Lysosome co-localization experiment of PGd@tNBs nanoparticles entering cells (Scale 5 μm). **(C)** Quantitative assessment of PGd@tNBs nanoparticle content within cells over 6–72 h. **(D)** Characterization of intracellular reactive oxygen species using DCFH-DA probe (Scale 5 μm). **(E)** Quantitative analysis of intracellular reactive oxygen species (*n* = 3). **(F)** Quantitative analysis of intracellular GSH content (*n* = 3). **(G)** UV-visible light characterization of intracellular SOD content. **(H)** Characterization of cell viability using Calcein-AM/PI staining (Scale 10 μm). **(I)** Quantitative analysis of cell viability after different treatments (*n* = 3). **(J)** Assessment of cell apoptosis after different treatments. **(K)** Quantitative analysis of cell apoptosis. **(L)** Cell count through Transwell chambers after different treatments (Scale 5 μm). **(M)** Quantitative analysis of cell count passing through Transwell chambers (*n* = 3). **(N)** Wound healing assay after different treatments (Scale 20 μm). **(O)** Quantitative assessment of wound healing after different treatments (*n* = 3). **(P)** SEM characterization of PGd@NBs nanoparticles binding to cells and subjected to ultrasound stimulation

The fundamental principle underpinning SDT revolves around the generation of ROS and their consequential biological effects [44]. Notably, piezoelectric materials can amplify ROS production under ultrasound, thereby enhancing the therapeutic effectiveness of SDT [10, 44–46]. Hydroxyl radicals (·OH) constitute the most reactive entities among ROS components, characterized by an extraordinarily high reaction rate constant of 10^9 with neighboring molecules upon their formation, thereby inducing cellular damage [47]. Ultrasound-triggered titanium dioxide generates ·OH and augments Fenton-like reactions [48]. Under ultrasound excitation, an increase in the concentration of PGd@tNBs nanoparticles significantly enhances the generation of ROS and ·OH (Fig. 6A). Under ultrasound exposure, PGd@tNBs nanoparticles induce surface charges, oxidizing GSH into GSSH and thus diminishing the antioxidant capacity of the solution. This phenomenon mirrors ultrasound-activated barium titanate, which generates charges to enhance enzyme activity and expedite GSH consumption [49]. In alignment with these findings, the concentration of GSH decreased proportionally with increasing concentrations of PGd@tNBs particles (Figure S14).

To characterize the sonodynamic therapeutic effects of PGd@tNBs nanoparticles on SK-BR3 cells, we assessed the time point at which these nanoparticles entered the cells. At 24 h of co-incubation between the nanoparticles and cells, a noticeable fluorescence co-localization phenomenon occurred (Fig. 6B). This indicates that a significant number of nanoparticles penetrated the cells within 24 h (Figures S15 and S16). Additionally, with the extension of co-incubation time, the quantity of nanoparticles entering the cells increased (Fig. 6C). Therefore, in the subsequent characterization of SDT efficacy, a series of experiments was conducted after co-incubation of nanoparticles with cells for 24 h.

Here, we define the group receiving ultrasound stimulation after the binding of nanoparticles to cells as PGd@tNBs + US1. The group receiving ultrasound stimulation after nanoparticles enter the cells is defined as PGd@tNBs + US2. The group where nanoparticles bind to cells, enter the cells, and receive repeated ultrasound stimulation is defined as PGd@tNBs + US1 + 2.

Mild stimulation can induce the generation of intracellular ROS (Fig. 6D), while PGd@tNBs nanoparticles further increase the generation of intracellular ROS under ultrasound (Fig. 6E). Interestingly, the cell activity of the PGd@tNBs + US1 + 2 group was the lowest, and the decrease in activity was most significant with time (Figure S17). At the same time, the relative generation of ROS showed a linear increase with time (Figure S18 and Figure S19). Subsequently, the PGd@tNBs + US1 + 2 group demonstrated the most substantial decrease in GSH content, a difference that was statistically significant when compared to both the PGd@tNBs + US1 and PGd@tNBs + US2 groups (Fig. 6F and Figure S20). Importantly, cellular constituents were extracted for analysis. The superoxide dismutase (SOD) content and reduced GSH content of the different treatment groups exhibited distinct degrees of reduction (Fig. 6G). Significantly, the PGd@tNBs + US1 + 2 group exhibited notably higher efficacy in SDT compared to both the PGd@tNBs + US1 and PGd@tNBs + US2 groups. Observing through Calcein-AM/PI staining, it was noted that the PGd@tNBs + US1 + 2 group exhibited a higher number of dead cells (Fig. 6H), and the cell viability in the PGd@tNBs + US1 + 2 group was lower compared to the PGd@tNBs + US1 and PGd@tNBs + US2 groups (Fig. 6I and Figure S21).

Solely repeating ultrasound stimulation prompted apoptosis in cells, with a specific emphasis on early apoptosis (Fig. 6J). When PGd@tNBs were either adhered to the cell membrane or had penetrated the cells before ultrasound stimulation, approximately 35% of cells underwent apoptosis. After a 24-hour cycle of repeated ultrasound stimulation, roughly 80% of cells underwent apoptosis, reflecting a substantial 45% escalation in the apoptosis rate (Fig. 6K). Concurrently, PGd@tNBs particles exhibited the ability to curb the invasive potential (Fig. 6L) and migratory capacity (Fig. 6M) of SK-BR3 breast cancer cells under ultrasound stimulation. At the 48-hour mark, the count of cells that traversed the Transwell chamber in the PGd@tNBs + US1 and PGd@tNBs + US2 groups approximated 50 cells, whereas the PGd@tNBs + US1 + 2 group exhibited a mere 15 cells (Fig. 6N). Moreover, the migration rate of the PGd@tNBs + US1 + 2 group was only 12.48 ± 1.11%, notably lower than that observed in the PGd@tNBs + US1 and PGd@tNBs + US2 groups, signifying a significant statistical distinction (Fig. 6O). Even after the ultrasound treatment, the distribution of PGd@tNBs nanoparticles on the cell surface remained more discernible than that of PGd@NBs nanoparticles (Fig. 6P and Figure S22).

### Anti-tumor therapeutic efficacy of PGd@NBs nanoparticles in vivo

**Fig. 7:**
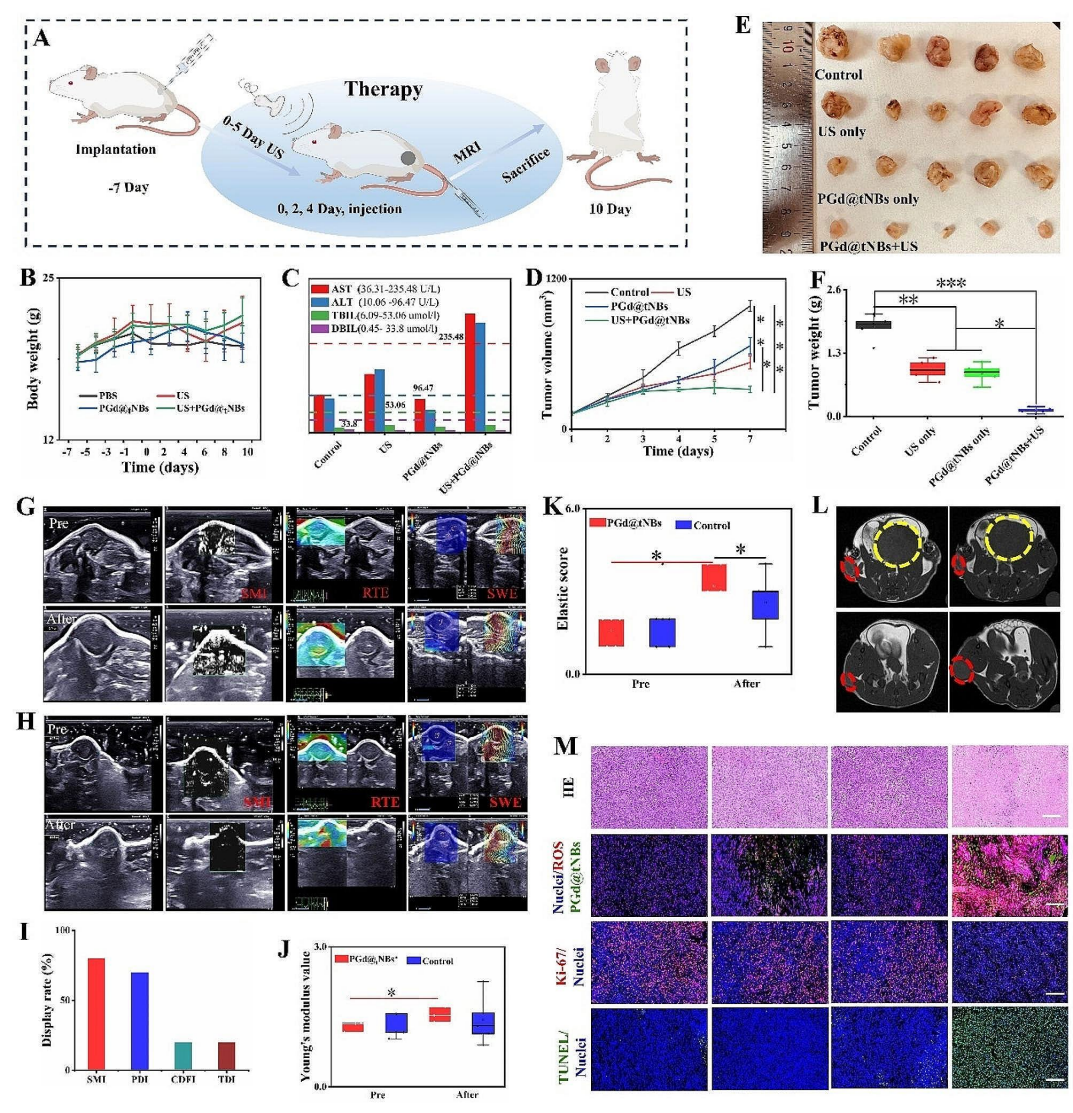
Anti-tumor therapeutic efficacy of PGd@NBs nanoparticles in vivo. **(A)** Schematic representation of Balb/c mice treatment and imaging. **(B)** Changes in body weight of mice in different groups. **(C)** Liver function results of mice in different groups. **(D)** Changes in tumor growth in different groups of mice. **(E)** Macroscopic images of tumor tissues from different groups of mice. **(F)** Tumor weights of mice in different groups. **(G)** and **(H)** Ultrasound blood flow imaging and ultrasound elastography of tumor tissues before and after treatment. **(I)** Ultrasound blood flow imaging display rate of mouse tumors. **(J)** Young’s modulus values before and after treatment. **(K)** Ultrasound elastography scores before and after treatment. **(L)** MRI imaging showing changes in tumors before and after treatment. **(M)** The ROS levels, Ki-67 proliferation, apoptotic status, distribution of PGd@tNBs within tumors, and H&E staining of tumor tissues after different treatments (Scale 20 μm)

Confirmed at the cellular level, based on the effectiveness of PGd@tNBs nanoparticle therapy, we conducted animal experiments to further verify. Figure 7A elucidates the schematic delineating the process from mouse treatment to euthanization. As the treatment regimen progressed, the PGd@tNBs + US group displayed slower tumor volume growth in comparison to the control group or the groups treated with ultrasound or PGd@tNBs nanoparticle independently. These distinctions were statistically significant (Fig. 7D). Furthermore, the tumor weight within the PGd@tNBs + US group was notably lower than that observed in the control group (Fig. 7E and F). Upon contrasting the ultrasound parameters of tumor tissues before and after treatment, it became evident that within the control group, the tumor tissue exhibited a significant increase in size and augmented blood flow on the two-dimensional images (Fig. 7G). In contrast, within the PGd@tNBs + US group, the tumor tissue’s size remained relatively unchanged and displayed a substantial reduction in blood flow signals (Fig. 7H). Collectively, the display rate of blood flow within the tumor tissue diminished (Fig. 7I). This reduction could potentially be attributed to the occlusion of tumor blood vessels due to inflammation post-treatment, consequently leading to a decreased nutrient supply to the tumor cells.

Post-treatment, the tumor tissue within the PGd@tNBs + US group demonstrated a remarkable elevation in hardness, with increase in elasticity score was particularly marked, displaying a significant divergence from the control group post-treatment (Fig. 7J). The Young’s modulus value manifesting a statistically significant distinction from that observed pre-treatment (Fig. 7K). The alterations in the tumor tissue post-treatment were visually captured through MRI (Fig. 7L). While individual treatments involving ultrasound stimulation or PGd@tNBs particles in isolation contributed to elevated levels of ROS within the tumor tissue, it was within the PGd@tNBs + US group that the highest ROS content was observed. Furthermore, PGd@tNBs particles exhibited proficient accumulation within the tumor region, characterized by relatively uniform distribution. Throughout the entire course of treatment, the mice’s body weight exhibited no significant fluctuations (Fig. 7B). The renal functions of the mice were maintained within the normal range (Table S1). With regard to liver function indicators, both AST and ALT levels were elevated beyond the normal range in the PGd@tNBs + US group (Fig. 7C). This phenomenon can be attributed to the primary metabolism of PGd@tNBs occurring within the liver and kidneys, leading to some degree of liver involvement. On the 1st, 3rd, and 5th days of the treatment regimen, blood routine tests were conducted through retroorbital venous blood collection. Notably, the PGd@tNBs + US group displayed an upsurge in lymphocyte and red blood cell count on the 5th day, concurrently with a decrease in mean corpuscular hemoglobin concentration (MCHC) and mean corpuscular hemoglobin (MCH) (Figure S23 and Table S2). Concurrently, pivotal organs of the mice were subjected to H&E staining, which unveiled that PGd@tNBs particles failed to induce substantial toxic effects on the heart, liver, spleen, lungs, or kidneys of the mice (Figure S24).

Concurrently, an evaluation of the proliferation and apoptosis status of the tumor tissue was conducted (Fig. 7M). Immunohistochemical staining targeting Ki-67, a recognized marker of cell proliferation, revealed a substantial reduction in cell proliferation within the PGd@tNBs + US group. The TUNEL assay similarly corroborated this observation, with the PGd@tNBs + US group exhibiting the highest count of apoptotic cells, while apoptosis induced by ultrasound in isolation or PGd@tNBs particles on their own remained minimal. H&E staining visually illustrated a reduction in the number of tumor cells, accompanied by pronounced nuclear condensation and fragmentation, discernible within the PGd@tNBs + US group. These findings harmonized with the outcomes of cellular experiments, steadfastly affirming the outstanding anti-tumor effects attributed to PGd@tNBs particles.

## Conclusion

In conclusion, the developed PGd@tNBs nanoparticles showcase exceptional stability, excellent biocompatibility, and notable piezoelectric properties. Upon exposure to ultrasound stimulation, these particles generate charge alterations that modify cell membrane potential, thereby instigating Ca^2+^ influx, mitochondrial membrane potential reduction, and the initiation of apoptotic processes. Furthermore, upon particle internalization and subsequent ultrasound stimulation, the ensuing ROS production amplifies tumor cell impairment. Consequently, the application of ultrasound-induced electrical stimulation through PGd@tNBs nanoparticles augments their role as potent sonosensitizers, effectively triggering SDT. This piezoelectric/sonodynamic therapy approach has proven effective in suppressing HER2 BC cell proliferation and promoting apoptosis. Significantly, the PGd@tNBs nanoparticles we synthesized possess both ultrasound imaging and MRI imaging capabilities, this functionality significantly enhances their clinical applicability, as it allows for the complementary utilization of the two imaging modalities, effectively overcoming their individual limitations.

### Electronic supplementary material

Below is the link to the electronic supplementary material.

Supplementary Material 1

## Data Availability

No datasets were generated or analysed during the current study.

## Abbreviations

SDT: Sonodynamic therapy
HER2 BC: HER2-positive breast cancer
P(VDF-TrFE): Poly (vinylidene fluoride-trifluoroethylene)
TME: Tumor microenvironment
CEUS: Contrast-enhanced ultrasound
MRI: Magnetic resonance imaging
ROS: Reactive oxygen species
DPPC: 1, 2-Dipalmitoyl-sn-glycero-3-phosphorylcholine
DSPE-PEG-Mal: 1, 2-distearoyl-sn-glycero-3-phosphoethanolamine-N-[maleimide(polyethyleneglycol)
DSPE-PEG-FITC: 1, 2-distearoyl-sn-glycero-3-phosphoethanolamine-poly(ethylene glycol)-Fluorescein
SOD: Superoxide Dismutase
GSH: Glutathione
XRD: X-ray diffraction analysis
XPS: X-ray Photoelectron Spectroscopy
PFM: Piezoresponse Force Microscopy
DLS: Dynamic Light Scattering
SEM: Scanning Electron Microscopy
TEM: Transmission Electron Microscopy
EDS: Energy-Dispersive X-ray Spectroscopy
CLSM: Confocal Laser Scanning Microscopy
SAI: Spectral Amplitude Imaging
CBC: Complete Blood Count
HE: Hematoxylin and Eosin
SMI: Superb Microvascular Imaging
TDI: Tissue Doppler Imaging
PDI: Power Doppler Imaging
TUNEL: Transferase dUTP nick end labeling
