# Supplementary material for: Ultrasonic-responsive piezoelectric stimulation enhances sonodynamic therapy for HER2-positive breast cancer
Source: J Nanobiotechnology. 2024 Jun 25;22:369. doi: 10.1186/s12951-024-02639-6 (PMC11197237; doi:10.1186/s12951-024-02639-6)
Supplement: Supplementary file 1 — Supplementary Material 1 [file 12951_2024_2639_MOESM1_ESM.docx]

Supplementary information

**Ultrasonic-Responsive Piezoelectric Stimulation Enhances Sonodynamic Therapy for HER2-Positive Breast Cancer**

Zhiguang Chen^1,2^, Lizhi Yang^1,2^, Zhimin Yang^2,3^, Zihua Wang^2,3^*, Wen He ^1^*, Wei Zhang^1^*

1. Department of ultrasound, Beijing Tiantan Hospital, Capital Medical University, Beijing 100050, China.
2. CAS Key Laboratory for Biomedical Effects of Nanomaterials and Nanosafety, CAS Center for Excellence in Nanoscience, National Center for Nanoscience and Technology, Beijing 100190, China.

3. Fujian Provincial Key Laboratory of Brain Aging and Neurodegenerative Diseases, School of Basic Medical Sciences, Fujian Medical University, Fuzhou 350122, Fujian, China.

**Corresponding authors:** E-mail: wangzh@iccas.ac.cn, hewen@bjtth.org, [ultrazw@sina.com](mailto:ultrazw@sina.com)





**Figure S1.** XPS of PGd NPs.


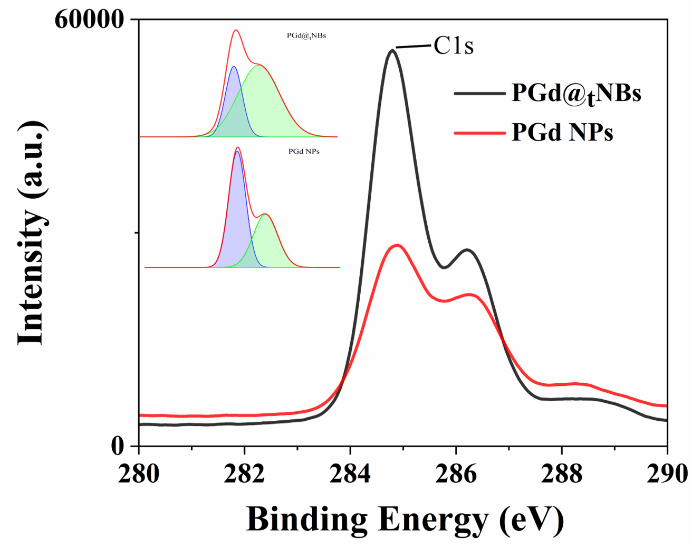


**Figure S2.** The valence state of element C in PGd NPs and PGd@tNBs.


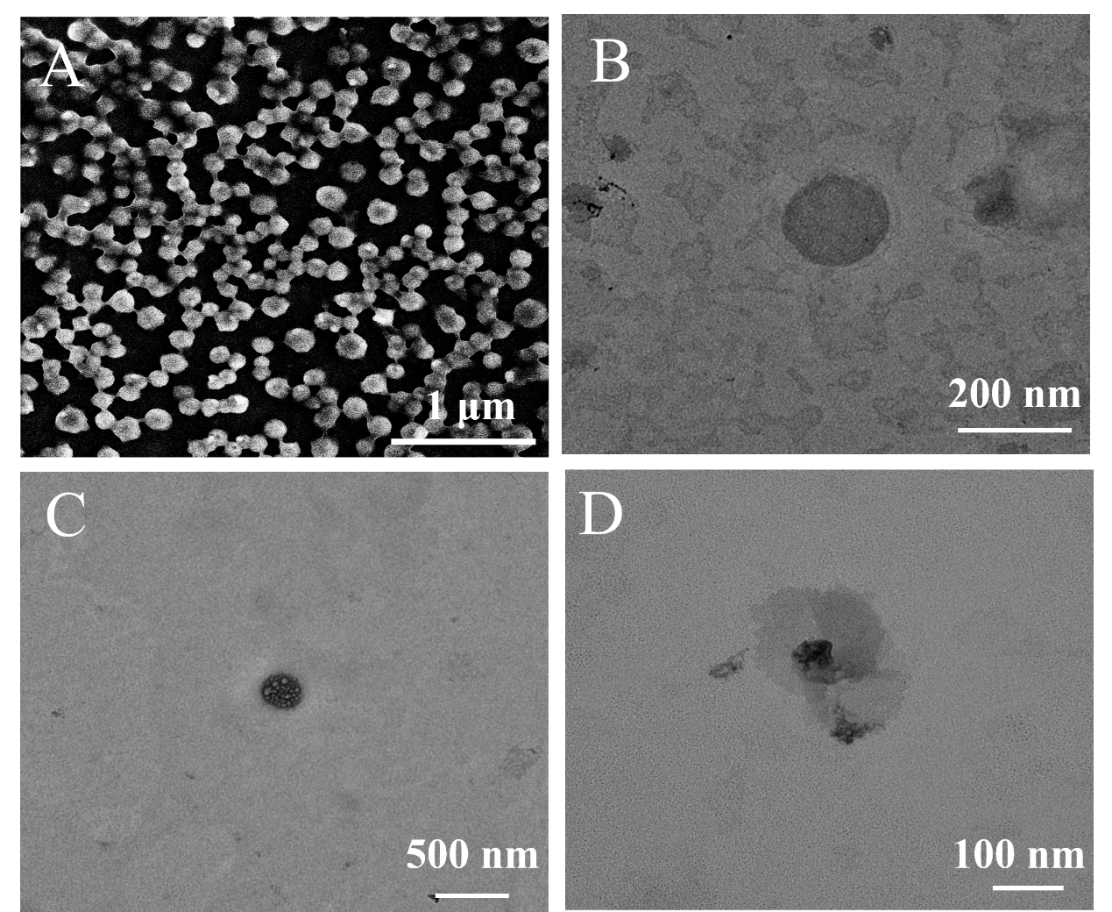


**Figure S3.** A) SEM of lipid shell. B) TEM of lipid shell. C) TEM of blending of PGd NPs with C_6_F_14_. [D) TEM of PGd@tNBs](mailto:B.PGd@tNBs).


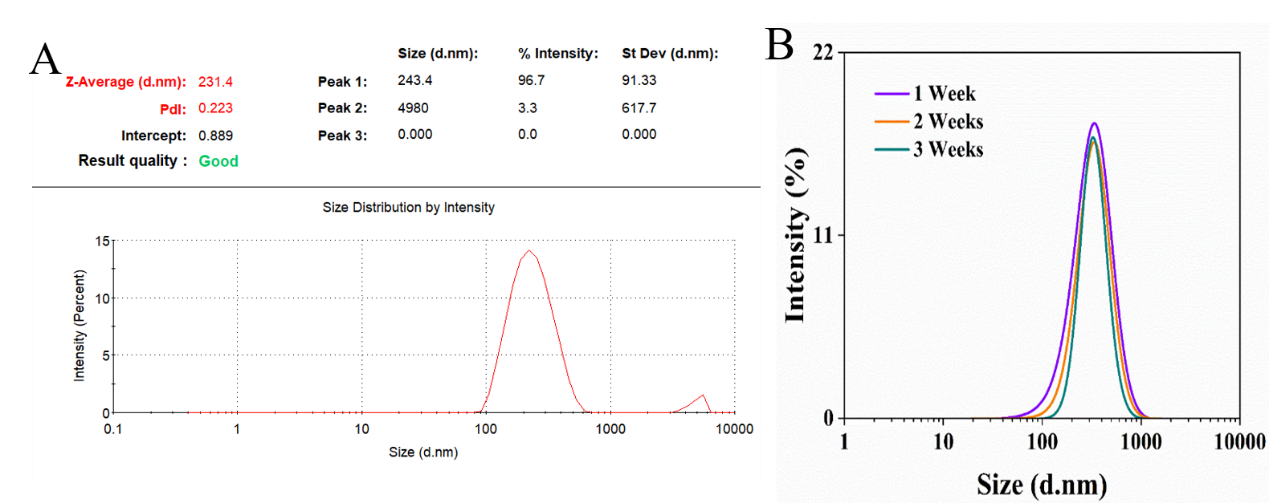


**Figure S4.** A) Polydispersity index (PDI) of PGd@tNBs particles. B) Particle size in the first, second, and third weeks after synthesis.


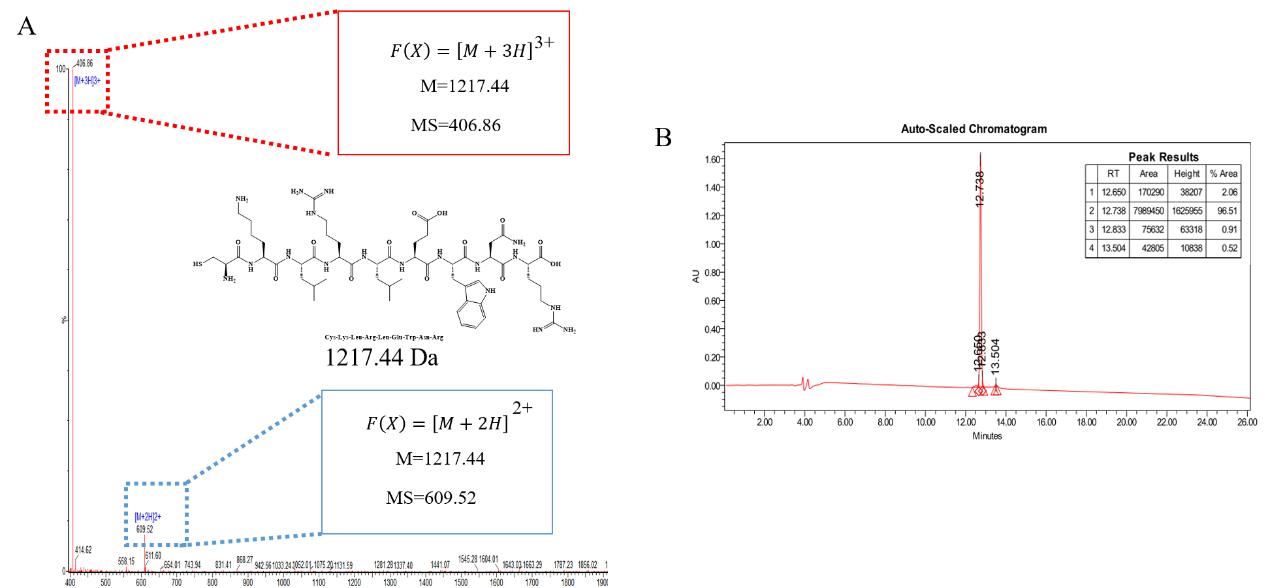
**Figure S5.** Molecular weight characterization of HER2 targeted peptide (CKLRLEWNR).


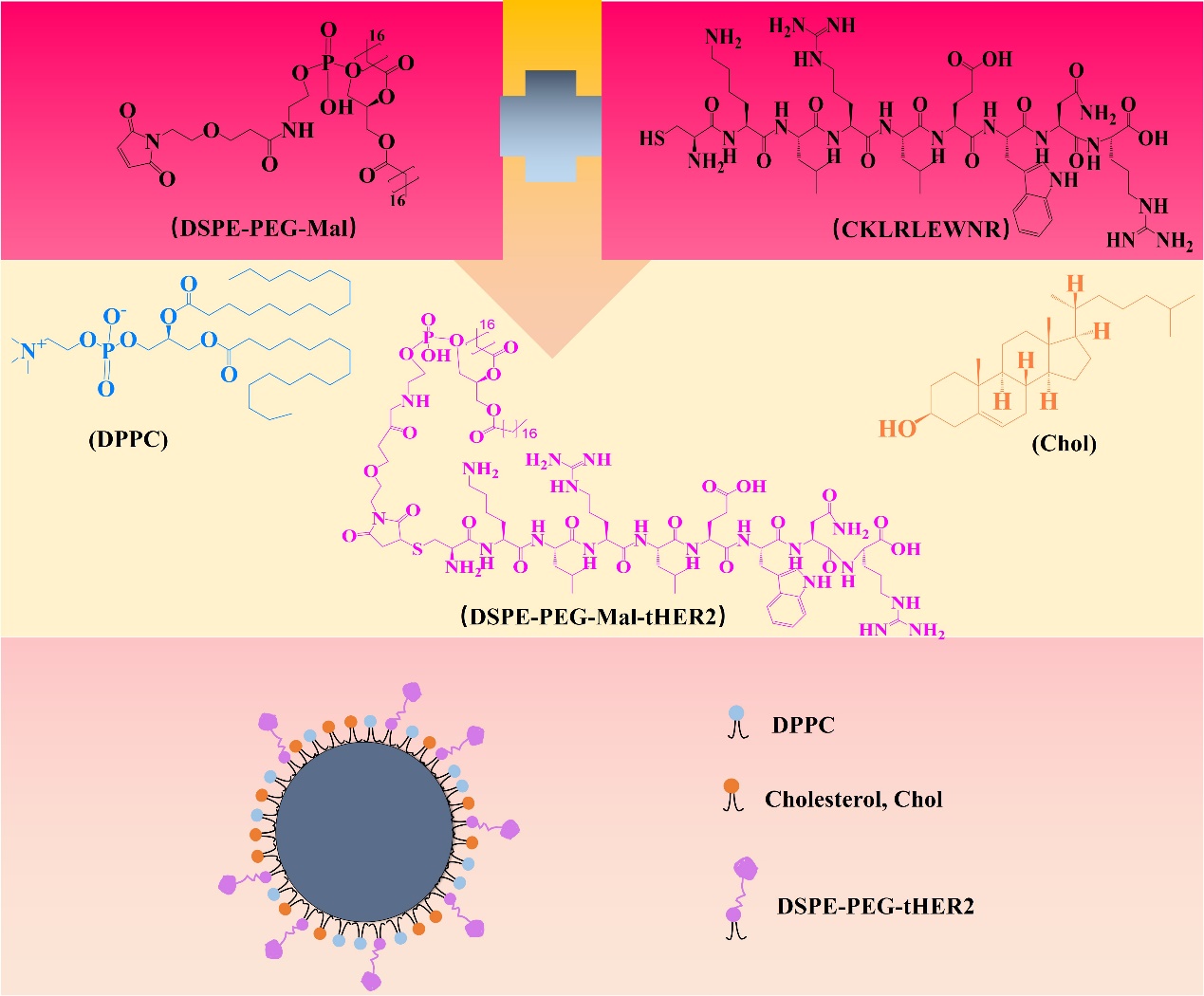


**Figure S6.** Molecular structure formula and schematic diagram of lipid shell.


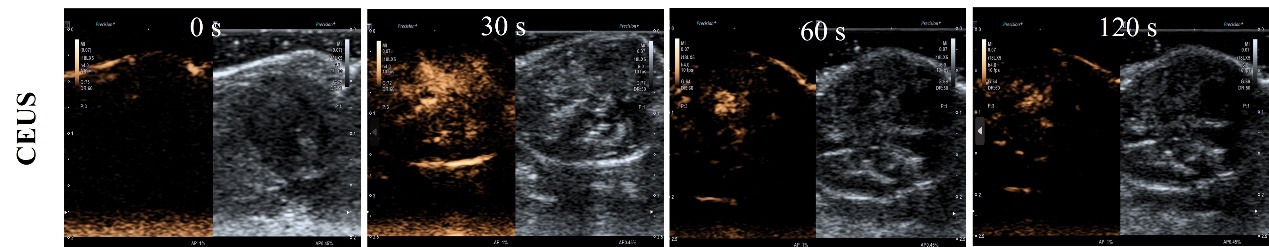


**Figure S7.** CEUS of PGd@tNBs in vivo tumor.





**Figure S8.** Detect the amplitude changes of P (VDF-TrFE) particles before and after reshaping through PFM. Estimate the piezoelectric coefficient *d*_33_ (*d*_33_ ≈ amplitude/voltage).


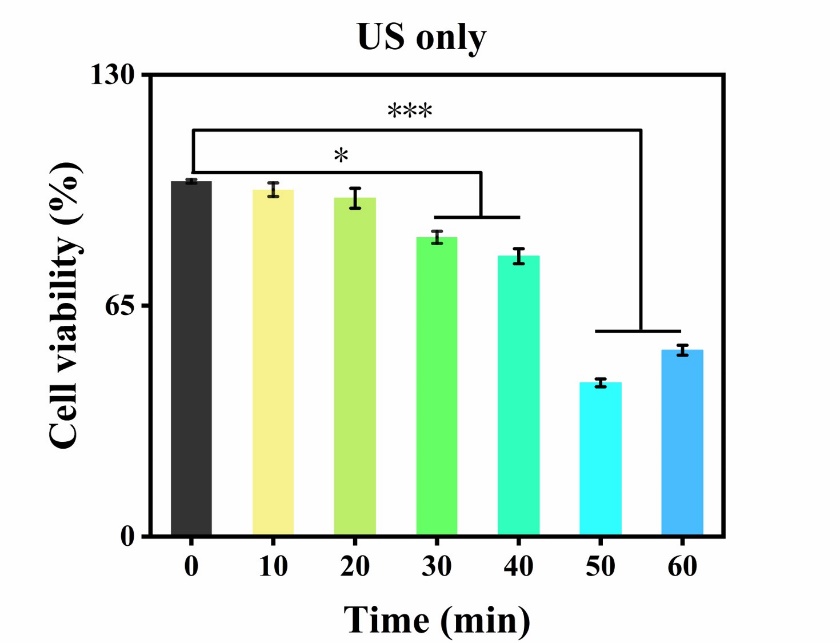


**Figure S9.** Relationship between ultrasound stimulation time and cell viability for MCF-7 cells (n=3).


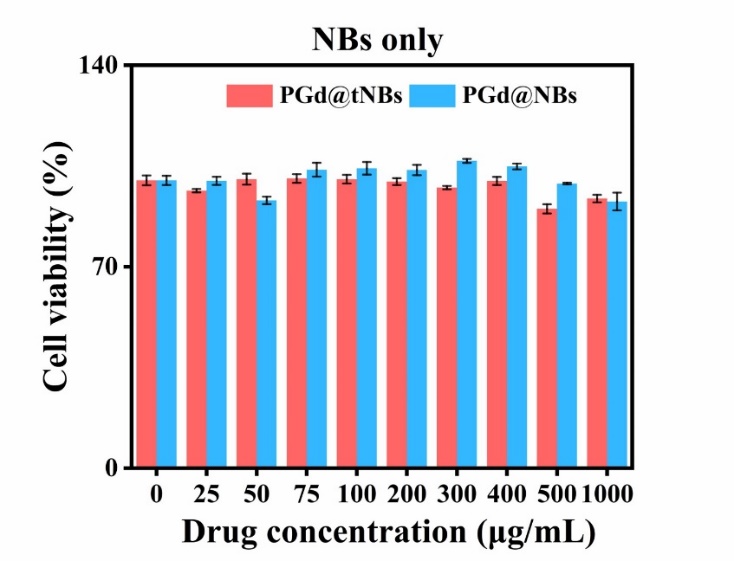


**Figure S10.** Impact of PGd@tNBs nanoparticles and PGd@NBs nanoparticles at different concentrations on MCF-7 (n=3).





**Figure S11.** Influence of PGd@tNBs nanoparticles and PGd@NBs nanoparticles at different concentrations on SK-BR3 cell viability under ultrasound stimulation (n=3).
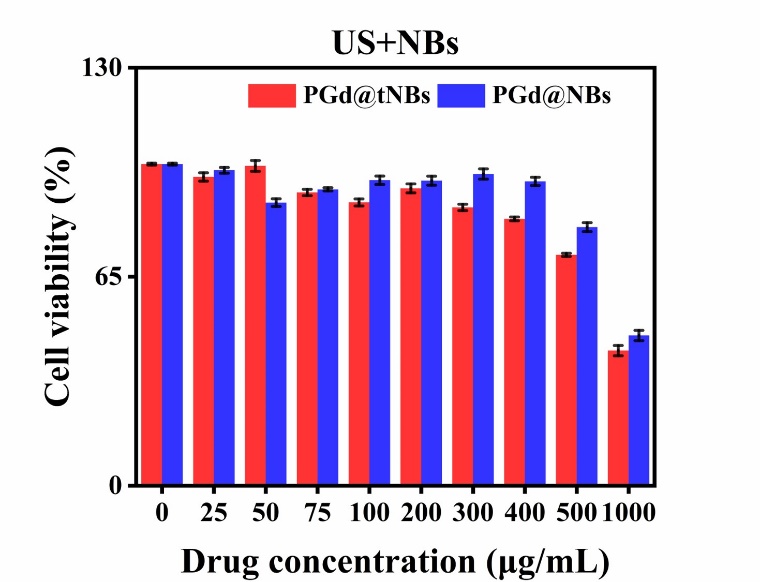


**Figure S12.** Influence of PGd@tNBs nanoparticles and PGd@NBs nanoparticles at different concentrations on MCF-7 cell viability under ultrasound stimulation (n=3).


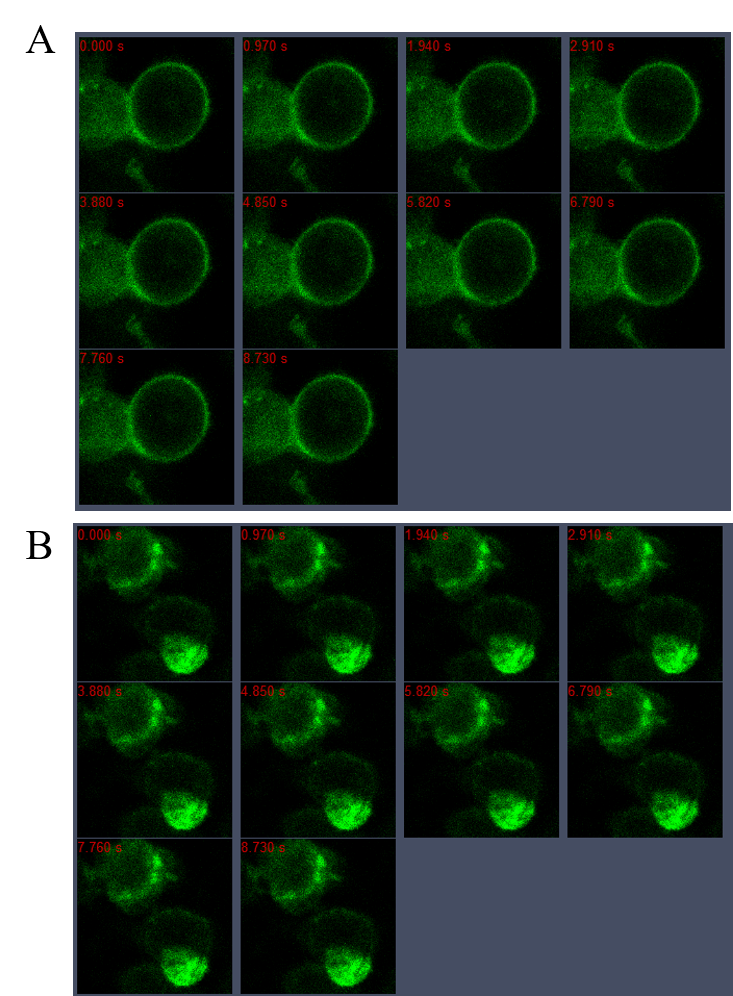


**Figure S13.** After staining the cells with FluoVolt^TM^ probe, the fluorescence intensity of the cell membrane was continuously detected for 10 seconds. A) Control group; B)PGd@tNBs +US Group.


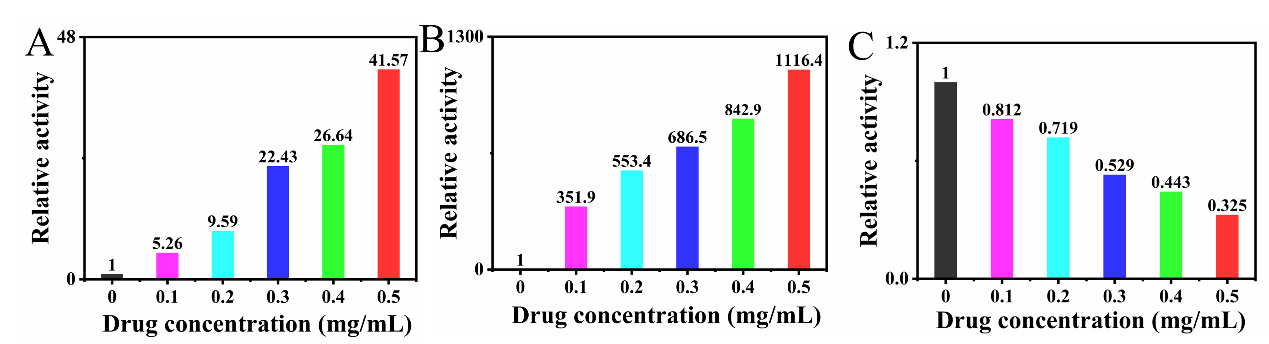


**Figure S14.** SDT capacity characterization of PGd@tNBs nanoparticles. **A)** Quantitative assessment of generated ROS. **B)** Quantitative assessment of generated ·OH. **C)** Quantitative assessment of GSH depletion.


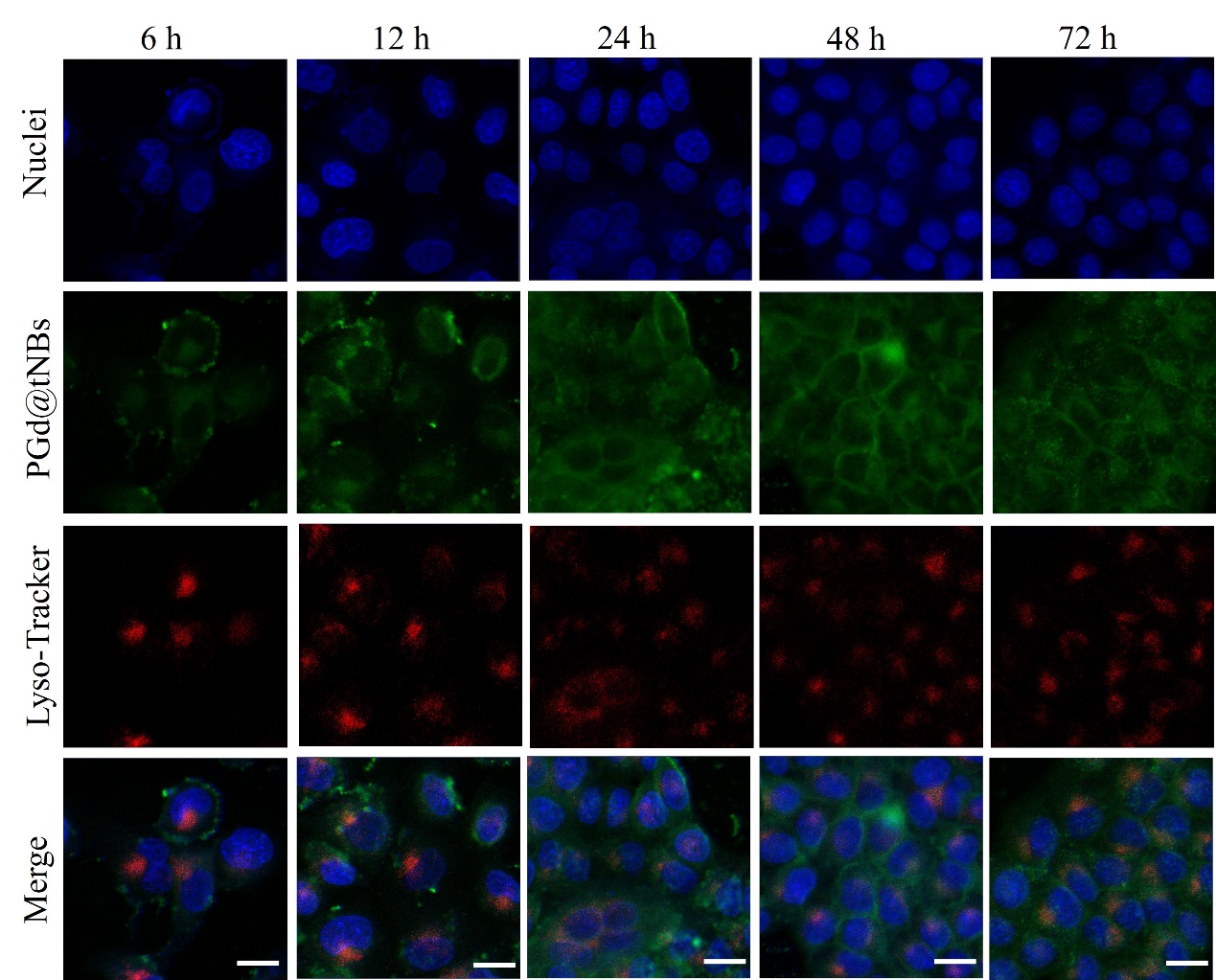


**Figure S15.** Characterization by CLSM when particles and cells are co incubated for 6-72 hours (Scale, 5 μm).


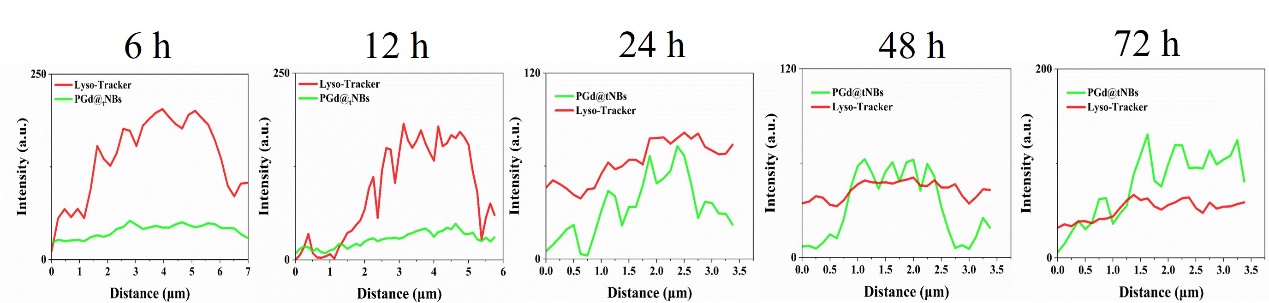


**Figure S16.** Fluorescence intensity analysis of particles and cells are co incubated for 6-72 hours.





**Figure S17.** Detection of cell activity over time under different treatment conditions using CCK8. ①: Control group; ②: US1 group; ③: US2 group; ④: PGd@tNBs Only group; ⑤: PGd@tNBs+US1 group; ⑥: PGd@tNBs+US2 group; ⑦ PGd@tNBs+US1+2 group.





**Figure S18.** Detection of changes in cellular ROS generation over time under different treatment conditions. ①: Control group; ②: US1 group; ③: US2 group; ④: PGd@tNBs Only group; ⑤: PGd@tNBs+US1 group; ⑥: PGd@tNBs+US2 group; ⑦ PGd@tNBs+US1+2 group.


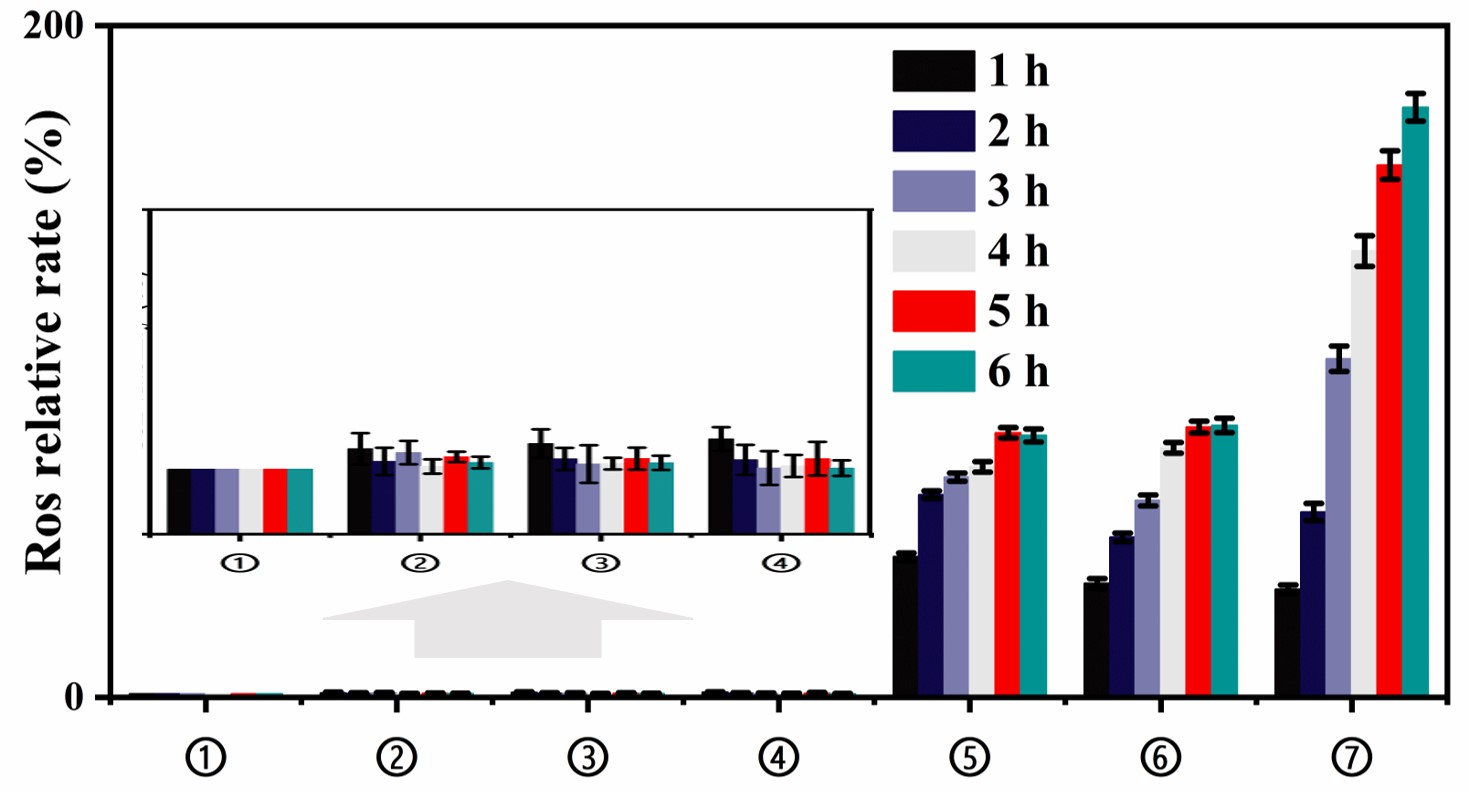


**Figure S19.** The relative amount of ROS generated by different treatment groups at different times. ①: Control group; ②: US1 group; ③: US2 group; ④: PGd@tNBs Only group; ⑤: PGd@tNBs+US1 group; ⑥: PGd@tNBs+US2 group; ⑦ PGd@tNBs+US1+2 group.


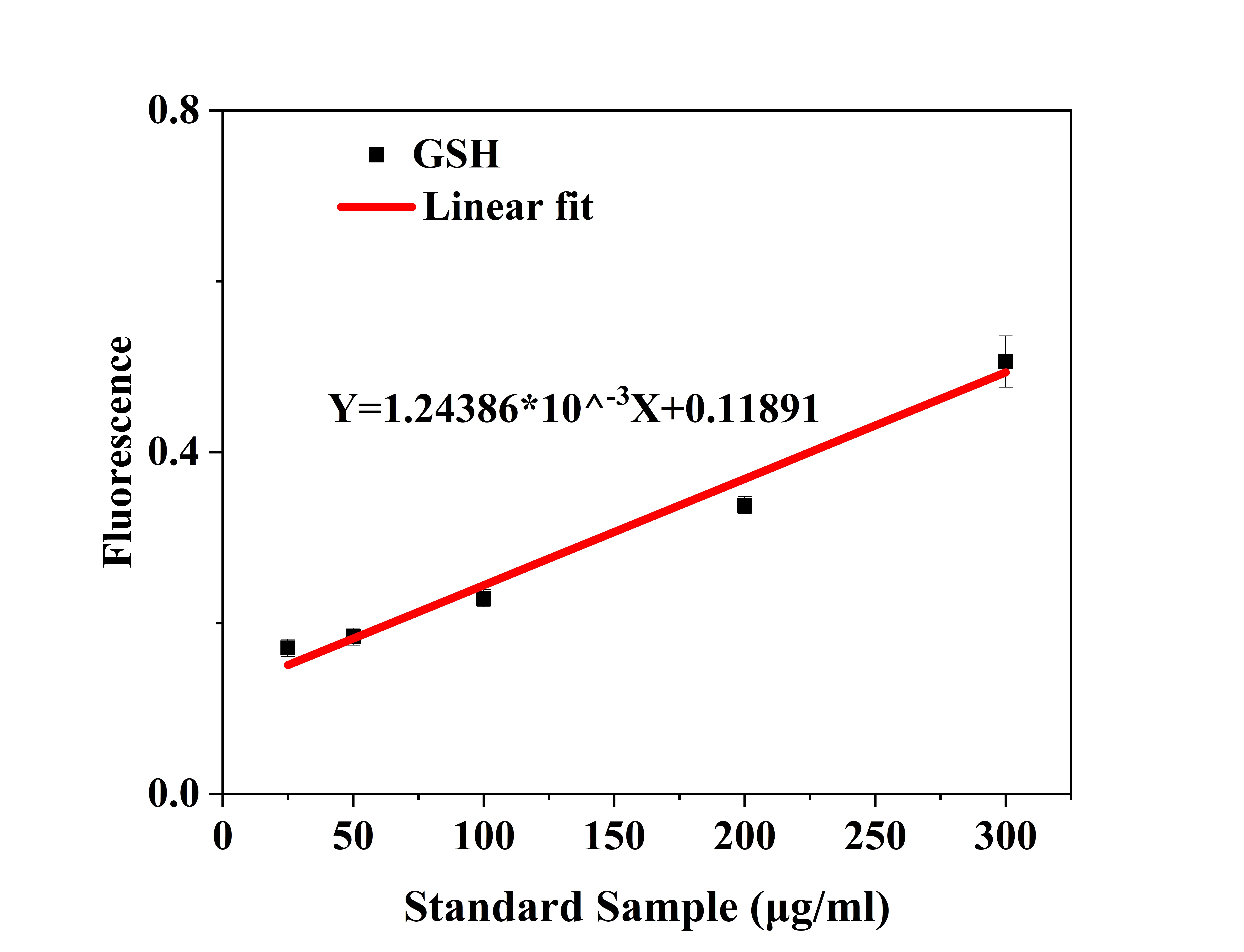


**Figure S20.** Standard curve for GSH.


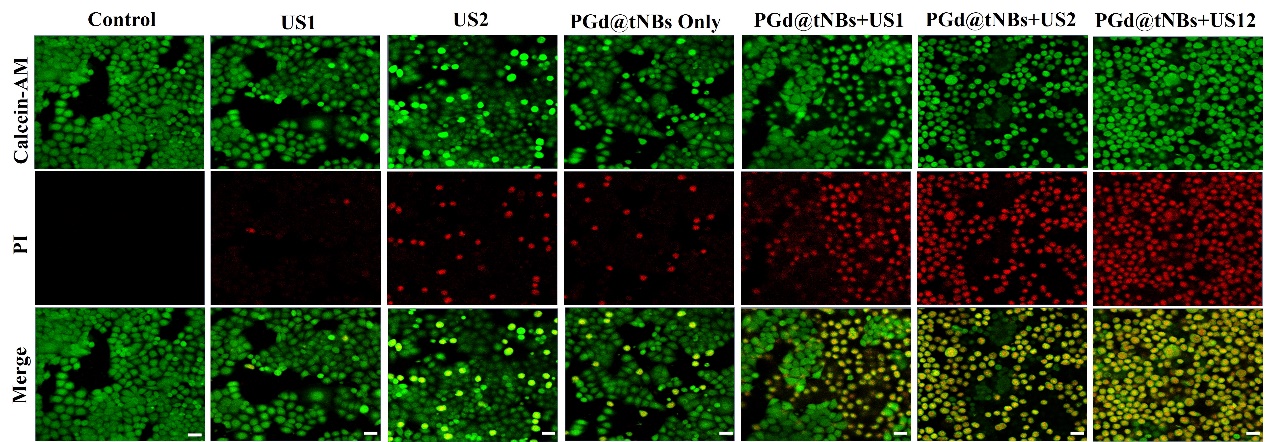


**Figure S21.** Detection of cell viability under different treatment conditions using the Calcein-AM/PI kit (Scale 10μm).


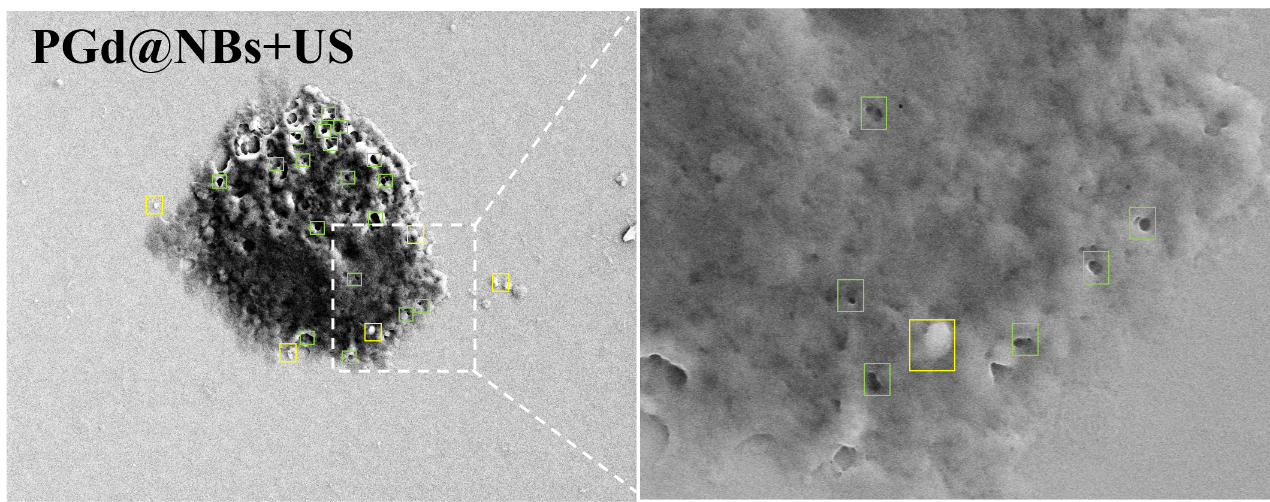


**Figure S22.** SEM characterization of PGd@tNBs nanoparticles binding to cells and subjected to ultrasound stimulation.


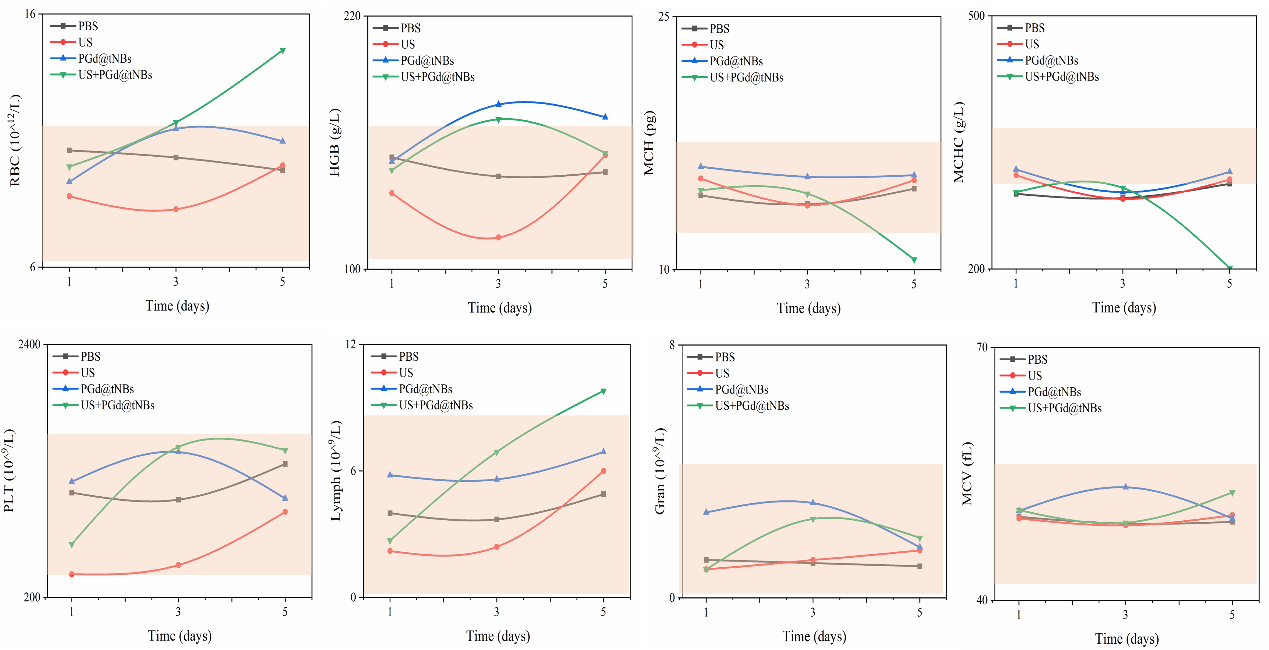


**Figure S23.** Changes in blood routine indicators on the 1st, 3rd, and 5th days of treatment in mice.


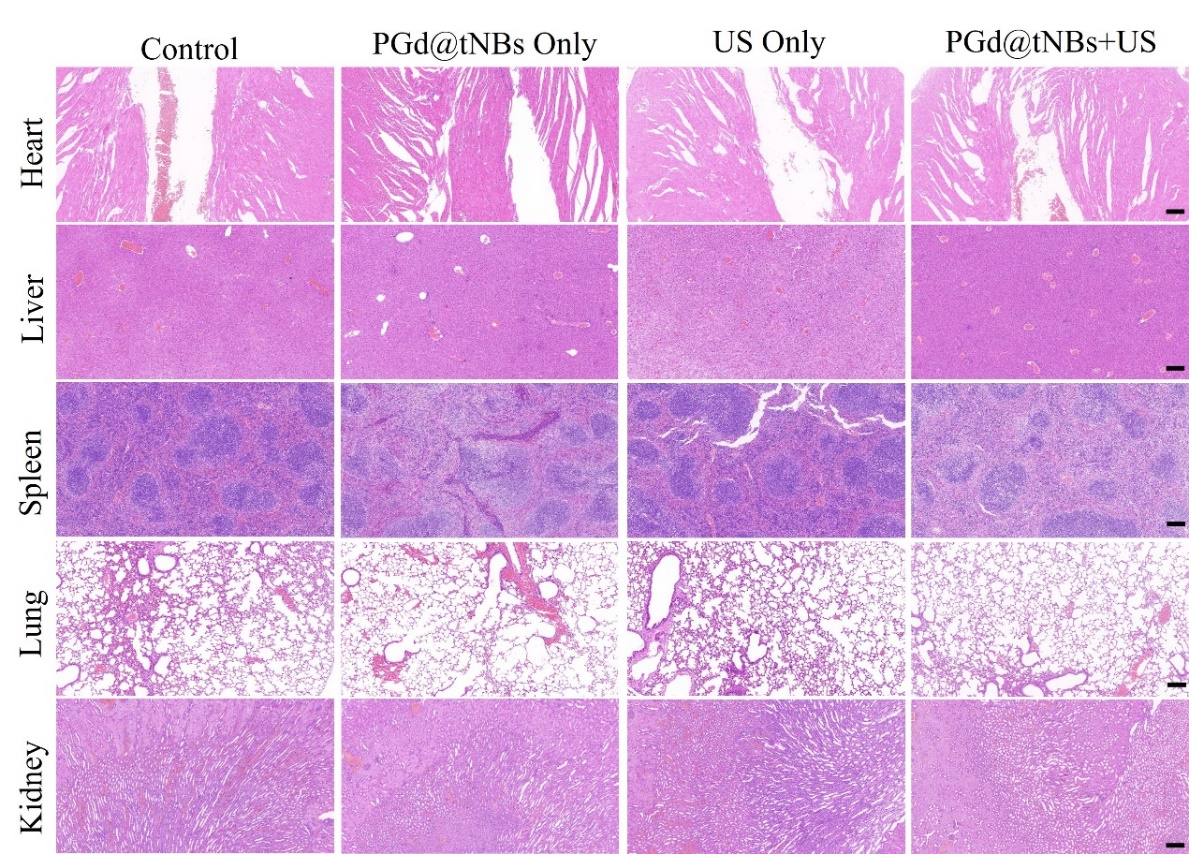


**Figure S24.** H&E staining of mouse heart, liver, spleen, lungs, and kidneys after different. Scale 100 μm.

**Table S1.** Biochemical examination of mouse blood during treatment.

|  | Control | US only | [PGd@tNBs only](mailto:PGd@tNBs%20only) | [PGd@tNBs+US](mailto:PGd@tNBs+US) | Normal range |
| --- | --- | --- | --- | --- | --- |
| ALT | 88.252 | 142.702 | 44.126 | 285.404 | 10.06-96.47U/L |
| AST | 86.370 | 151.703 | 97.462 | 308.458 | 36.31-235.48U/L |
| TBIL | 15.055 | 19.236 | 11.854 | 19.128 | 6.09-53.06umol/L |
| DBIL | 5.546 | 6.599 | 7.716 | 6.402 | 0.45-33.89umol/L |
| BUN | 16.453 | 13.622 | 11.967 | 16.436 | 10.81-34.74mg/dL |
| UA | 27.395 | 33.008 | 35.03 | 37.05 | 44.42-224.77umol/L |

**Table S2.** Mouse blood routine examination during treatment.

| **Group** | Day 1 | Day 3 | Day 5 | Normal range | |
| --- | --- | --- | --- | --- | --- |
| [**PGd@tNBs+US**](mailto:PGd@tNBs+US) |  |  |  |  |  |
| Lymph# | 2.7 | 6.9 | 9.8 | 0.6-8.9 | 10^9/L |
| Gran# | 0.9 | 2.5 | 1.9 | 0.23-3.6 | 10^9/L |
| RBC | 9.97 | 11.72 | 14.57 | 6.5-11.5 | 10^12/L |
| HGB | 147 | 171 | 155 | 110-165 | g/L |
| MCV | 50.7 | 49.2 | 52.8 | 41-55 | fL |
| MCH | 14.7 | 14.5 | 10.6 | 13-18 | pg |
| MCHC | 291 | 296 | 201 | 300-360 | g/L |
| PLT | 662 | 1508 | 1480 | 400-1600 | 10^9/L |
| [**PGd@tNBs only**](mailto:PGd@tNBs%20only) |  |  |  |  |  |
| Lymph# | 5.8 | 5.6 | 6.9 | 0.6-8.9 | 10^9/L |
| Gran# | 2.7 | 3 | 1.6 | 0.23-3.6 | 10^9/L |
| RBC | 9.37 | 11.46 | 10.97 | 6.5-11.5 | 10^12/L |
| HGB | 151 | 178 | 172 | 110-165 | g/L |
| MCV | 50.6 | 53.4 | 49.7 | 41-55 | fL |
| MCH | 16.1 | 15.5 | 15.6 | 13-18 | pg |
| MCHC | 318 | 291 | 315 | 300-360 | g/L |
| PLT | 1206 | 1463 | 1060 | 400-1600 | 10^9/L |
| **Control** |  |  |  |  |  |
| Lymph# | 4 | 3.7 | 4.9 | 0.6-8.9 | 10^9/L |
| Gran# | 1.2 | 1.1 | 1 | 0.23-3.6 | 10^9/L |
| RBC | 10.61 | 10.33 | 9.83 | 6.5-11.5 | 10^12/L |
| HGB | 153 | 144 | 146 | 110-165 | g/L |
| MCV | 49.9 | 49.1 | 49.3 | 41-55 | fL |
| MCH | 14.4 | 13.9 | 14.8 | 13-18 | pg |
| MCHC | 289 | 284 | 301 | 300-360 | g/L |
| PLT | 1111 | 1050 | 1361 | 400-1600 | 10^9/L |
| **US only** |  |  |  |  |  |
| Lymph# | 2.2 | 2.4 | 6 | 0.6-8.9 | 10^9/L |
| Gran# | 0.9 | 1.2 | 1.5 | 0.23-3.6 | 10^9/L |
| RBC | 8.8 | 8.29 | 10.02 | 6.5-11.5 | 10^12/L |
| HGB | 136 | 115 | 154 | 110-165 | g/L |
| MCV | 49.7 | 48.9 | 50.1 | 41-55 | fL |
| MCH | 15.4 | 13.8 | 15.3 | 13-18 | pg |
| MCHC | 311 | 283 | 306 | 300-360 | g/L |
| PLT | 401 | 481 | 943 | 400-1600 | 10^9/L |
